# Dietary Supplements and Nutraceuticals under Investigation for COVID-19 Prevention and Treatment

**DOI:** 10.1128/mSystems.00122-21

**Published:** 2021-05-04

**Authors:** Ronan Lordan, Halie M. Rando, COVID-19 Review Consortium, Vikas Bansal, Casey S. Greene

**Affiliations:** a Institute for Translational Medicine and Therapeutics, Perelman School of Medicine, University of Pennsylvania, Philadelphia, Pennsylvania, USA; b Department of Systems Pharmacology and Translational Therapeutics, University of Pennsylvania, Philadelphia, Pennsylvania, USA; c Department of Biochemistry and Molecular Genetics, University of Colorado School of Medicine, Aurora, Colorado, USA; d Center for Health AI, University of Colorado School of Medicine, Aurora, Colorado, USA; e Childhood Cancer Data Lab, Alex’s Lemonade Stand Foundation, Philadelphia, Pennsylvania, USA; University of California San Diego

**Keywords:** COVID-19, review, SARS-CoV-2, vitamin D, nutraceuticals

## Abstract

Coronavirus disease 2019 (COVID-19) has caused global disruption and a significant loss of life. Existing treatments that can be repurposed as prophylactic and therapeutic agents may reduce the pandemic’s devastation. Emerging evidence of potential applications in other therapeutic contexts has led to the investigation of dietary supplements and nutraceuticals for COVID-19. Such products include vitamin C, vitamin D, omega 3 polyunsaturated fatty acids, probiotics, and zinc, all of which are currently under clinical investigation. In this review, we critically appraise the evidence surrounding dietary supplements and nutraceuticals for the prophylaxis and treatment of COVID-19. Overall, further study is required before evidence-based recommendations can be formulated, but nutritional status plays a significant role in patient outcomes, and these products may help alleviate deficiencies. For example, evidence indicates that vitamin D deficiency may be associated with a greater incidence of infection and severity of COVID-19, suggesting that vitamin D supplementation may hold prophylactic or therapeutic value. A growing number of scientific organizations are now considering recommending vitamin D supplementation to those at high risk of COVID-19. Because research in vitamin D and other nutraceuticals and supplements is preliminary, here we evaluate the extent to which these nutraceutical and dietary supplements hold potential in the COVID-19 crisis.

**IMPORTANCE** Sales of dietary supplements and nutraceuticals have increased during the pandemic due to their perceived “immune-boosting” effects. However, little is known about the efficacy of these dietary supplements and nutraceuticals against the novel coronavirus (severe acute respiratory syndrome coronavirus 2 [SARS-CoV-2]) or the disease that it causes, CoV disease 2019 (COVID-19). This review provides a critical overview of the potential prophylactic and therapeutic value of various dietary supplements and nutraceuticals from the evidence available to date. These include vitamin C, vitamin D, and zinc, which are often perceived by the public as treating respiratory infections or supporting immune health. Consumers need to be aware of misinformation and false promises surrounding some supplements, which may be subject to limited regulation by authorities. However, considerably more research is required to determine whether dietary supplements and nutraceuticals exhibit prophylactic and therapeutic value against SARS-CoV-2 infection and COVID-19. This review provides perspective on which nutraceuticals and supplements are involved in biological processes that are relevant to recovery from or prevention of COVID-19.

## INTRODUCTION

The year 2020 saw scientists and the medical community scrambling to repurpose or discover novel host-directed therapies against the coronavirus (CoV) disease 2019 (COVID-19) pandemic caused by the spread of the novel *Severe acute respiratory syndrome-related coronavirus 2* (SARS-CoV-2). This rapid effort led to the identification of some promising pharmaceutical therapies for hospitalized patients, such as remdesivir and dexamethasone. Furthermore, most societies have adopted nonpharmacological preventative measures, such as utilizing public health strategies that reduce the transmission of SARS-CoV-2. However, during this time, many individuals sought additional protections via the consumption of various dietary supplements and nutraceuticals that they believed to confer beneficial effects. While a patient’s nutritional status does seem to play a role in COVID-19 susceptibility and outcomes (1–5), the beginning of the pandemic saw sales of vitamins and other supplements soar despite a lack of any evidence supporting their use against COVID-19. In the United States, for example, dietary supplement and nutraceutical sales have shown modest annual growth in recent years (approximately 5%, or a $345 million increase in 2019), but during the 6-week period preceding 5 April 2020, they increased by 44% ($435 million) relative to the same period in 2019 (6). While growth subsequently leveled off, sales continued to boom, with a further 16% ($151 million) increase during the 6 weeks preceding 17 May 2020 relative to 2019 (6). In France, New Zealand, India, and China, similar trends in sales were reported (7–10). The increase in sales was driven by a consumer perception that dietary supplements and nutraceuticals would protect consumers from infection and/or mitigate the impact of infection due to the various “immune-boosting” claims of these products (11, 12).

Due to the significant interest from the general public in dietary additives, whether and to what extent nutraceuticals or dietary supplements can provide any prophylactic or therapeutic benefit remain topics of interest for the scientific community. Nutraceuticals and dietary supplements are related but distinct nonpharmaceutical products. Nutraceuticals are classified as supplements with health benefits beyond their basic nutritional value (13, 14). The key difference between a dietary supplement and a nutraceutical is that nutraceuticals should not only supplement the diet but also aid in the prophylaxis and/or treatment of a disorder or disease (15). However, dietary supplements and nutraceuticals, unlike pharmaceuticals, are not subject to the same regulatory protocols that protect consumers of medicines. Indeed, nutraceuticals do not entirely fall under the responsibility of the Food and Drug Administration (FDA), but they are monitored as dietary supplements according to the Dietary Supplement, Health and Education Act of 1994 (DSHEA) (16) and the Food and Drug Administration Modernization Act of 1997 (FDAMA) (17). Due to increases in sales of dietary supplements and nutraceuticals, in 1996 the FDA established the Office of Dietary Supplement Programs (ODSP) to increase surveillance. Novel products or nutraceuticals must now submit a new dietary ingredient notification to the ODSP for review. There are significant concerns that these legislations do not adequately protect the consumer, as they ascribe responsibility to the manufacturers to ensure the safety of the product before manufacturing or marketing (18). Manufacturers are not required to register or even seek approval from the FDA to produce or sell food supplements or nutraceuticals. Health or nutrient content claims for labeling purposes are approved based on an authoritative statement from the Academy of Sciences or relevant federal authorities once the FDA has been notified and on the basis that the information is known to be true and not deceptive (18). Therefore, there is often a gap between perceptions by the American public about a nutraceutical or dietary supplement and the actual clinical evidence surrounding its effects.

Despite differences in regulations, similar challenges exist outside the United States. In Europe, the safety of supplements is monitored by the European Union (EU) under Directive 2002/46/EC (19). However, nutraceuticals are not directly mentioned. Consequently, nutraceuticals can be generally described as either a medicinal product under Directive 2004/27/EC (20) or as a foodstuff under Directive 2002/46/EC of the European Council. In order to synchronize the various existing legislations, Regulation EC 1924/2006 on nutrition and health claims (21) was put into effect to assure customers of the safety and efficacy of products and to deliver understandable information to consumers. However, specific legislation for nutraceuticals is still elusive. Health claims are permitted on a product label only following compliance with the European Food Safety Authority (EFSA) guidelines on nutrition and health claims and authorization from EFSA (22, 250). EFSA does not currently distinguish between food supplements and nutraceuticals for health claim applications of new products, as claim authorization is dependent on the availability of clinical data in order to substantiate efficacy (23). These guidelines seem to provide more protection to consumers than the FDA regulations but potentially at the cost of innovation in the sector (24). The situation becomes even more complicated when comparing regulations at a global level, as countries such as China and India have existing regulatory frameworks for traditional medicines and phytomedicines not commonly consumed in Western society (25). Currently, there is debate among scientists and regulatory authorities surrounding the development of a widespread regulatory framework to deal with the challenges of safety and health claim substantiation for nutraceuticals (18, 23), as these products do not necessarily follow the same rigorous clinical trial frameworks used to approve the use of pharmaceuticals. Such regulatory disparities have been highlighted by the pandemic, as many individuals and companies have attempted to profit from the vulnerabilities of others by overstating claims in relation to the treatment of COVID-19 using supplements and nutraceuticals. The FDA has written several letters to prevent companies marketing or selling products based on false hyperbolic promises about preventing SARS-CoV-2 infection or treating COVID-19 (26–28). These letters came in response to efforts to market nutraceutical prophylactics against COVID-19, some of which charged the consumer as much as $23,000 (29). There have even been some incidents highlighted in the media because of their potentially life-threatening consequences; for example, the use of oleandrin was touted as a potential “cure” by individuals close to the former president of the United States despite its high toxicity (30). Thus, heterogeneous and at times relaxed regulatory standards have permitted high-profile cases of the sale of nutraceuticals and dietary supplements that are purported to provide protection against COVID-19, despite a lack of research into these compounds.

Notwithstanding the issues of poor safety, efficacy, and regulatory oversight, some dietary supplements and nutraceuticals have exhibited therapeutic and prophylactic potential. Some have been linked with reduced immunopathology, antiviral, and anti-inflammatory activities or even the prevention of acute respiratory distress syndrome (ARDS) (11, 31, 32). A host of potential candidates that target various aspects of the COVID-19 viral pathology have been highlighted in the literature, while others are thought to prime the host immune system. These candidates include vitamins and minerals along with extracts and omega-3 polyunsaturated fatty acids (n-3 PUFA) (33). *In vitro* and *in vivo* studies suggest that nutraceuticals containing phycocyanobilin, *N*-acetylcysteine, glucosamine, selenium, or phase 2 inductive nutraceuticals (e.g., ferulic acid, lipoic acid, or sulforaphane) can prevent or modulate RNA virus infections via amplification of the signaling activity of mitochondrial antiviral-signaling (MAVS) protein and activation of Toll-like receptor 7 (34). Phase 2 inductive molecules used in the production of nutraceuticals are known to activate nuclear factor erythroid 2-related factor 2 (Nrf2), which is a protein regulator of antioxidant enzymes that leads to the induction of several antioxidant enzymes, such as gamma-glutamylcysteine synthetase. While these compounds appear promising, further animal and human studies are required to assess the therapeutic potential of these various nutrients and nutraceuticals against COVID-19. For the purpose of this review, we have highlighted some of the main dietary supplements and nutraceuticals that are under investigation for their potential prophylactic and therapeutic applications. These include n-3 PUFA, zinc, vitamins C and D, and probiotics.

## n-3 PUFA

One category of supplements that have been explored for beneficial effects against various viral infections is the n-3 PUFAs (33), commonly referred to as omega-3 fatty acids, which include eicosapentaenoic acid (EPA) and docosahexaenoic acid (DHA). EPA and DHA intake can come from a diet high in fish or through dietary supplementation with fish oils or purified oils (35). Other, more sustainable sources of EPA and DHA include algae (36, 37), which can also be exploited for their rich abundance of other bioactive compounds, such as angiotensin-converting enzyme inhibitor peptides and antiviral agents, including phycobiliproteins, sulfated polysaccharides, and calcium-spirulan (38). n-3 PUFAs have been investigated for many years for their therapeutic potential (39). Supplementation with fish oils is generally well tolerated (39), and intake of n-3 PUFAs through dietary sources or supplementation is specifically encouraged for vulnerable groups, such as pregnant and lactating women (40, 41). As a result, these well-established compounds have drawn significant interest for their potential immune effects and therapeutic potential.

Particular interest has arisen in n-3 PUFAs as potential therapeutics against diseases associated with inflammation. n-3 PUFAs have been found to modulate inflammation by influencing processes such as leukocyte chemotaxis, adhesion molecule expression, and the production of eicosanoids (42, 43). This and other evidence indicate that n-3 PUFAs may have the capacity to modulate the adaptive immune response (14, 35, 42); for example, they have been found to influence antigen presentation and the production of CD4^+^ Th1 cells, among having other relevant effects (44). Certainly, preliminary evidence from banked blood samples from 100 COVID-19 patients suggests that patients with a higher omega-3 index, a measure of the amount of EPA and DHA in red blood cells, had a lower risk of death due to COVID-19 (45). Interest has also arisen as to whether nutritional status related to n-3 PUFAs can also affect inflammation associated with severe disease, such as ARDS or sepsis (46, 47). ARDS and sepsis hold particular concern in the treatment of severe COVID-19; an analysis of 82 deceased COVID-19 patients in Wuhan, China, during January to February 2020 reported that respiratory failure (associated with ARDS) was the cause of death in 69.5% of cases and that sepsis or multiorgan failure accounted for 28.0% of deaths (48). Research in ARDS prior to the current pandemic suggests that n-3 PUFAs may hold some therapeutic potential. One study randomized 16 consecutive ARDS patients to receive either a fish oil-enriched lipid emulsion or a control lipid emulsion (comprised of 100% long-chain triglycerides) under a double-blind design (49). They reported a statistically significant reduction in leukotriene B4 levels in the group receiving the fish oil-enriched emulsion, suggesting that the fish oil supplementation may have reduced inflammation. However, they also reported that most of their tests were not statistically significant, and therefore it seems that additional research using larger sample sizes is required. A recent meta-analysis of 10 randomized controlled trials (RCTs) examining the effects of n-3 PUFAs on ARDS patients did not find evidence of any effect on mortality, although the effect on secondary outcomes could not be determined due to a low quality of evidence (50). However, another meta-analysis that examined 24 RCTs studying the effects of n-3 fatty acids on sepsis, including ARDS-induced sepsis, did find support for an effect on mortality when n-3 fatty acids were administered via enteral nutrition, although a paucity of high-quality evidence again limited conclusions (51). Therefore, despite theoretical support for an immunomodulatory effect of n-3 PUFAs in COVID-19, evidence from existing RCTs is insufficient to determine whether supplementation offers an advantage in a clinical setting that would be relevant to COVID-19.

Another potential mechanism that has led to interest in n-3 PUFAs as protective against viral infections, including COVID-19, is their potential as precursor molecules for the biosynthesis of endogenous specialized proresolving mediators (SPM), such as protectins and resolvins, that actively resolve inflammation and infection (52). SPM have exhibited beneficial effects against a variety of lung infections, including some caused by RNA viruses (53, 54). Several mechanisms for SPM have been proposed, including preventing the release of proinflammatory cytokines and chemokines or increasing the phagocytosis of cellular debris by macrophages (55). In influenza, SPM promote antiviral B lymphocytic activities (56), and protectin D1 has been shown to increase survival from H1N1 viral infection in mice by affecting the viral replication machinery (57). It has thus been hypothesized that SPM may aid in the resolution of the cytokine storm and pulmonary inflammation associated with COVID-19 (58, 59). Another theory is that some comorbidities, such as obesity, lead to deficiencies of SPM, which may in turn be related to the occurrence of adverse outcomes for COVID-19 (60). However, not all studies are in agreement that n-3 PUFAs or their resulting SPM are effective against infections (61). At a minimum, the effectiveness of n-3 PUFAs against infections would be dependent on the dosage, timing, and the specific pathogens responsible (62). On another level, there is still the question of whether fish oils can raise the levels of SPM levels upon ingestion and in response to acute inflammation in humans (63). Currently, Karolinska University Hospital is running a trial that will measure the levels of SPM as a secondary outcome following intravenous supplementation of n-3 PUFAs in hospitalized COVID-19 patients to determine whether n-3 PUFAs provide therapeutic value (64, 65). Therefore, while this mechanism provides theoretical support for a role for n-3 PUFAs against COVID-19, experimental support is still needed.

A third possible mechanism by which n-3 PUFAs may benefit COVID-19 patients arises from the fact that some COVID-19 patients, particularly those with comorbidities, are at a significant risk of thrombotic complications, including arterial and venous thrombosis (66, 67). Therefore, the use of prophylactic and therapeutic anticoagulants and antithrombotic agents is under consideration (68, 69). Considering that there is significant evidence that n-3 fatty acids and other fish oil-derived lipids possess antithrombotic properties and anti-inflammatory properties (35, 70, 71), they may have therapeutic value against the prothrombotic complications of COVID-19. In particular, concerns have been raised within the medical community about using investigational therapeutics on COVID-19 patients who are already on antiplatelet therapies due to preexisting comorbidities because the introduction of such therapeutics may lead to issues with dosing and drug choice and/or negative drug-drug interactions (68). In such cases, dietary sources of n-3 fatty acids or other nutraceuticals with antiplatelet activities may hold particular value for reducing the risk of thrombotic complications in patients already receiving pharmaceutical antiplatelet therapies. A new clinical trial (72) is currently recruiting COVID-19-positive patients to investigate the anti-inflammatory activity of a recently developed, highly purified nutraceutical derivative of EPA known as icosapent ethyl (Vascepa) (73). Other randomized controlled trials that are in the preparatory stages intend to investigate the administration of EPA and other bioactive compounds to COVID-19-positive patients in order to observe whether anti-inflammatory effects or disease state improvements occur (74, 75). Finally, while there have been studies investigating the therapeutic value of n-3 fatty acids against ARDS in humans, there is still limited evidence of their effectiveness (76). It should be noted that the overall lack of human studies in this area means that there is limited evidence as to whether these supplements may affect COVID-19 infection. Consequently, the clinical trials that are under way and those that have been proposed will provide valuable insight into whether the anti-inflammatory potential of n-3 PUFAs and their derivatives can be beneficial to the treatment of COVID-19. All the same, while the evidence is not present to draw conclusions about whether n-3 PUFAs will be useful in treating COVID-19, there is likely little harm associated with a diet rich in fish oils, and interest in n-3 PUFA supplementation by the general public is unlikely to have negative effects.

## ZINC

Zinc is a nutrient supplement that may exhibit some benefits against RNA viral infections. Zinc is a trace metal obtained from dietary sources or supplementation and is important for the maintenance of immune cells involved in adaptive and innate immunity (77). Supplements can be administered orally as a tablet or as a lozenge and are available in many forms, such as zinc picolinate, zinc acetate, and zinc citrate. Zinc is also available from dietary sources, including meat, seafood, nuts, seeds, legumes, and dairy. The role of zinc in immune function has been extensively reviewed (77). Zinc is an important signaling molecule, and zinc levels can alter host defense systems. In inflammatory situations, such as an infection, zinc can regulate leukocyte immune responses and modulate the nuclear factor kappa–light-chain enhancer of activated B cells, thus altering cytokine production (78, 79). In particular, zinc supplementation can increase levels of natural killer cells, which are important cells for host defense against viral infections (77, 80). As a result of these immune-related functions, zinc is also under consideration for possible benefits against COVID-19.

Adequate zinc intake has been associated with a reduced incidence of infection (81) and antiviral immunity (82). A randomized, double-blind, placebo-controlled trial that administered zinc supplementation to elderly subjects over the course of a year found that zinc supplementation decreased susceptibility to infection and that zinc deficiency was associated with increased susceptibility to infection (81). Clinical trial data support the utility of zinc to diminish the duration and severity of symptoms associated with common colds when it is provided within 24 h of the onset of symptoms (83, 84). An observational study showed that COVID-19 patients had significantly lower zinc levels than those of healthy controls and that zinc-deficient COVID-19 patients (those with levels less than 80 μg/dl) tended to have more complications (70.4% versus 30.0%; *P = *0.009) and potentially prolonged hospital stays (7.9 versus 5.7 days; *P = *0.048) compared with those of patients who were not zinc deficient (85). In coronaviruses specifically, *in vitro* evidence has demonstrated that the combination of zinc (Zn^2+^) and zinc ionophores (pyrithione) can interrupt the replication mechanisms of SARS-CoV–green fluorescent protein (GFP) (a fluorescently tagged SARS-CoV-1) and a variety of other RNA viruses (86, 87). Currently, there are over 20 clinical trials registered with the intention of using zinc in a preventative or therapeutic manner for COVID-19. However, many of these trials proposed the use of zinc in conjunction with hydroxychloroquine and azithromycin (88–91), and it is not known how the lack of evidence supporting the use of hydroxychloroquine will affect the investigation of zinc. One retrospective observational study of New York University Langone hospitals in New York, NY, compared outcomes among hospitalized COVID-19 patients administered hydroxychloroquine and azithromycin with zinc sulfate (*n* = 411) versus hydroxychloroquine and azithromycin alone (*n* = 521). Notably, zinc is the only treatment that was used in this trial that is still under consideration as a therapeutic agent due to the lack of efficacy and potential adverse events associated with hydroxychloroquine and azithromycin against COVID-19 (92–94). While the addition of zinc sulfate did not affect the duration of hospitalization, the length of intensive care unit (ICU) stays, or the duration of patient ventilation, univariate analyses indicated that zinc did increase the frequency of patients discharged and decreased the requirement for ventilation, referrals to the ICU, and mortality (95). However, a smaller retrospective study at Hoboken University Medical Center New Jersey failed to find an association between zinc supplementation and the survival of hospitalized patients (96). Therefore, whether zinc contributes to COVID-19 recovery remains unclear. Other trials are now investigating zinc in conjunction with other supplements, such as vitamin C or n-3 PUFA (75, 97). Though there is, overall, encouraging data for zinc supplementation against the common cold and viral infections, there is currently limited evidence to suggest that zinc supplementation has any beneficial effects against the current novel COVID-19; thus, the clinical trials that are under way will provide vital information on the efficacious use of zinc in COVID-19 prevention and/or treatment. However, given the limited risk and the potential association between zinc deficiency and illness, maintaining a healthy diet to ensure an adequate zinc status may be advisable for individuals seeking to reduce their likelihood of infection.

## VITAMIN C

Vitamins B, C, D, and E have also been suggested as potential nutrient supplement interventions for COVID-19 (33, 98). In particular, vitamin C has been proposed as a potential therapeutic agent against COVID-19 due to its long history of use against the common cold and other respiratory infections (99, 100). Vitamin C can be obtained via dietary sources, such as fruits and vegetables, or via supplementation. Vitamin C plays a significant role in promoting immune function due to its effects on various immune cells. It affects inflammation by modulating cytokine production, decreasing histamine levels, enhancing the differentiation and proliferation of T- and B-lymphocytes, increasing antibody levels, and protecting against the negative effects of reactive oxygen species, among other effects related to COVID-19 pathology (101–103). Vitamin C is utilized by the body during viral infections, as evinced by lower concentrations in leukocytes and lower concentrations of urinary vitamin C. Postinfection, these levels return to baseline ranges (104–108). It has been shown that as little as 0.1 g/day of vitamin C can maintain normal plasma levels of vitamin C in healthy individuals, but higher doses of at least 1 to 3 g/day are required for critically ill patients in ICUs (109). Indeed, vitamin C deficiency appears to be common among COVID-19 patients (110, 111). COVID-19 is also associated with the formation of microthrombi and coagulopathy (112), which contribute to its characteristic lung pathology (113), but these symptoms can be ameliorated by early infusions of vitamin C to inhibit endothelial surface P-selectin expression and platelet-endothelial cell adhesion (114). Intravenous vitamin C also reduced D-dimer levels in a case study of 17 COVID-19 patients (115). D-dimer levels are an important indicator of thrombus formation and breakdown and are notably elevated in COVID-19 patients (116, 117). There is therefore preliminary evidence suggesting that vitamin C status and vitamin C administration may be relevant to COVID-19 outcomes.

Larger-scale studies of vitamin C, however, have provided mixed results. A recent meta-analysis found consistent support for regular vitamin C supplementation reducing the duration of the common cold but that supplementation with vitamin C (>200 mg) failed to reduce the incidence of colds (118). Individual studies have found vitamin C to reduce the susceptibility of patients to lower respiratory tract infections, such as pneumonia (119). Another meta-analysis demonstrated that in 12 trials, vitamin C supplementation reduced the length of stay of patients in ICUs by 7.8% (95% confidence interval [CI], 4.2% to 11.2%; *P = *0.00003). Furthermore, high doses (1 to 3 g/day) significantly reduced the length of an ICU stay by 8.6% in six trials (*P = *0.003). Vitamin C also shortened the duration of mechanical ventilation by 18.2% in three trials in which patients required intervention for over 24 h (95% CI, 7.7% to 27%; *P* = 0.001) (109). Despite these findings, an RCT of 167 patients known as CITRUS ALI failed to show a benefit of a 96-h infusion of vitamin C to treat ARDS (120). Clinical trials specifically investigating vitamin C in the context of COVID-19 have now begun, as highlighted by Carr and Rowe (100). These trials intend to investigate the use of intravenous vitamin C in hospitalized COVID-19 patients. The first trial to report initial results took place in Wuhan, China (121). These initial results indicated that the administration of 12 g of intravenous vitamin C/12 h for 7 days in 56 critically ill COVID-19 patients resulted in a promising reduction of 28-day mortality (*P = *0.06) in a univariate survival analysis (122). Indeed, the same study reported a significant decrease in interleukin 6 (IL-6) levels by day 7 of vitamin C infusion (*P = *0.04) (122). Additional studies that are being conducted in Canada, China, Iran, and the United States will provide additional insight into whether vitamin C supplementation affects COVID-19 outcomes on a larger scale.

Even though evidence supporting the use of vitamin C is beginning to emerge, we will not know how effective vitamin C is as a therapeutic for quite some time. Currently (as of January 2021), over 15 trials that either are recruiting, are active, or are currently in preparation are registered with ClinicalTrials.gov. When completed, these trials will provide crucial evidence on the efficacy of vitamin C as a therapeutic for COVID-19 infection. However, the majority of supplementation studies investigate the intravenous infusion of vitamin C in severely ill patients. Therefore, there is a lack of studies investigating the potential prophylactic administration of vitamin C via oral supplementation for healthy individuals or potentially asymptomatic SARS-CoV-2-positive patients. Once again, vitamin C intake is part of a healthy diet, and the vitamin likely presents minimal risk, but its potential prophylactic or therapeutic effects against COVID-19 are yet to be determined. To maintain vitamin C status, it would be prudent for individuals to ensure that they consume the recommended dietary allowance of vitamin C to maintain a healthy immune system (1). The recommended dietary allowance according to the FDA is 75 to 90 mg/day, whereas EFSA recommends 110 mg/day (124).

## VITAMIN D

Of all of the supplements under investigation, vitamin D has become a leading prophylactic and therapeutic candidate against SARS-CoV-2. Vitamin D can modulate both the adaptive and the innate immune system and is associated with various aspects of immune health and antiviral defense (125–129). Vitamin D can be sourced through diet or supplementation, but it is mainly biosynthesized by the body on exposure to UV light (UVB) from sunlight. Vitamin D deficiency is associated with an increased susceptibility to infection (130). In particular, vitamin D-deficient patients are at risk of developing acute respiratory infections (131) and ARDS (131). 1,25-Dihydroxyvitamin D3 is the active form of vitamin D that is involved in adaptive and innate responses; however, due to its low concentration and a short half-life of a few hours, vitamin D levels are typically measured by the longer-lasting and more abundant precursor 25-hydroxyvitamin D. The vitamin D receptor is expressed in various immune cells, and vitamin D is an immunomodulator of antigen-presenting cells, dendritic cells, macrophages, monocytes, and T- and B-lymphocytes (130, 132). Due to its potential immunomodulating properties, vitamin D supplementation may be advantageous to maintaining a healthy immune system.

Early in the pandemic, it was postulated that an individual’s vitamin D status may significantly affect their risk of developing COVID-19 (133). This hypothesis was derived from the fact that the current pandemic emerged in Wuhan, China, during winter, when 25-hydroxyvitamin D concentrations are at their lowest due to a lack of sunlight, whereas in the Southern Hemisphere, where it was nearing the end of the summer and 25-hydroxyvitamin D concentrations were higher, the number of cases was low. This led researchers to question whether there was a seasonal component to the SARS-CoV-2 pandemic and whether vitamin D levels might play a role (133–136). Though it is assumed that COVID-19 is seasonal, multiple other factors that can affect vitamin D levels should also be considered. These factors include an individual’s nutritional status, age, occupation, skin pigmentation, potential comorbidities, and exposure to sunlight, which varies due to latitude, among other factors. Indeed, it has been estimated that each degree of latitude north of 28 degrees corresponds to a 4.4% increase of COVID-19 mortality, indirectly linking a person’s vitamin D levels via exposure to UVB light to COVID-19 mortality (134).

As the pandemic has evolved, additional research of varying quality has investigated some of the potential links identified early in the pandemic (133) between vitamin D and COVID-19. Indeed, studies are beginning to investigate whether there is any prophylactic and/or therapeutic relationship between vitamin D and COVID-19. A study in Switzerland demonstrated that 27 SARS-CoV-2-positive patients exhibited 25-hydroxyvitamin D plasma concentrations that were significantly lower (11.1 ng/ml) than those of SARS-CoV-2-negative patients (24.6 ng/ml; *P = *0.004), an association that held when patients greater than 70 years old were stratified (137). These findings seem to be supported by a Belgian observational study of 186 SARS-CoV-2-positive patients exhibiting symptoms of pneumonia, for whom 25-hydroxyvitamin D plasma concentrations were measured and computed tomography (CT) scans of the lungs were obtained upon hospitalization (138). A significant difference in 25-hydroxyvitamin D levels was observed between the SARS-CoV-2 patients and 2,717 season-matched hospitalized controls. It is not clear from the study which diseases caused the control subjects to be admitted at the time of their 25-hydroxyvitamin D measurement, which makes it difficult to assess the observations reported. Both female and male patients possessed lower median 25-hydroxyvitamin D concentrations than the control group as a whole (18.6 ng/ml versus 21.5 ng/ml; *P = *0.0016) and a higher rate of vitamin D deficiency (58.6% versus 42.5%). However, when comparisons were stratified by sex, evidence of sexual dimorphism became apparent, as female patients had 25-hydroxyvitamin D levels that were equivalent to those of females in the control group, whereas male patients were deficient in 25-hydroxyvitamin D relative to levels in male controls (67% versus 49%; *P = *0.0006). Notably, vitamin D deficiency was progressively lower in males with advancing radiological disease stages (*P = *0.001). However, these studies are supported by several others that indicate that vitamin D status may be an independent risk factor for the severity of COVID-19 (139–142) and for COVID-19 patients relative to population-based controls (143). Indeed, serum concentrations of 25-hydroxyvitamin D above 30 ng/ml, which indicate vitamin D sufficiency, seems to be associated with a reduction in serum C-reactive protein, an inflammatory marker, along with increased lymphocyte levels, which suggests that vitamin D levels may modulate the immune response by reducing the risk for a cytokine storm in response to SARS-CoV-2 infection (143). Among severe COVID-19 cases in India, a high percentage of patients were vitamin D deficient, with 97% of patients observed to have low serum 25-hydroxyvitamin D levels (mean concentration, 6.2 ng/ml) compared to asymptomatic COVID-19 patients, among whom only 33% were vitamin D deficient (with a mean 25-hydroxyvitamin D concentration of 27.9 ng/ml) (144). In the same study, vitamin D deficiency was associated with higher levels of inflammatory markers, including IL-6, ferritin, and tumor necrosis factor alpha. Collectively, these studies add to a multitude of observational studies reporting potential associations between low levels of 25-hydroxyvitamin D and COVID-19 incidence and severity (137, 142, 143, 145–151).

Despite the large number of studies establishing a link between vitamin D status and COVID-19 severity, an examination of data from the UK Biobank did not support this thesis (152, 153). These analyses examined 25-hydroxyvitamin D concentrations alongside SARS-CoV-2 positivity and COVID-19 mortality in over 340,000 UK Biobank participants. However, these studies have caused considerable debate that will likely be settled following further studies (154, 155). Overall, while the evidence suggests that there is likely an association between low serum 25-hydroxyvitamin D and COVID-19 incidence, these studies must be interpreted with caution, as there is the potential for reverse causality, bias, and other confounding factors, including that vitamin D deficiency is also associated with numerous preexisting conditions and risk factors that can increase the risk for severe COVID-19 (1, 134, 156, 157).

While these studies inform us of the potential importance of vitamin D sufficiency and the risk of SARS-CoV-2 infection and severe COVID-19, they fail to conclusively determine whether vitamin D supplementation can therapeutically affect the clinical course of COVID-19. In one study, 40 vitamin D-deficient asymptomatic or mildly symptomatic patients were randomized to receive either 60,000 IU of cholecalciferol daily for at least 7 days (*n* = 16) or a placebo (*n* = 24), with a target serum 25-hydroxyvitamin D level of >50 ng/ml. At day 7, 10 patients achieved >50 ng/ml, followed by another 2 patients by day 14. By the end of the study, the treatment group had a greater proportion of vitamin D-deficient participants that tested negative for SARS-CoV-2 RNA, and they had a significantly lower fibrinogen levels, potentially indicating a beneficial effect (158). A pilot study in Spain determined that early administration of high-dose calcifediol (∼21,000 IU on days 1 and 2 and ∼11,000 IU on days 3 to 7 of hospital admission) with hydroxychloroquine and azithromycin to 50 hospitalized COVID-19 patients significantly reduced ICU admissions and may have reduced disease severity versus hydroxychloroquine and azithromycin alone (159). Although this study received significant criticism from the National Institute for Health and Care Excellence (NICE) in the United Kingdom (COVID-19 rapid evidence summary: vitamin D for COVID-19 [https://www.nice.org.uk/guidance/ng187/evidence/evidence-reviews-for-the-use-of-vitamin-d-supplementation-as-prevention-and-treatment-of-covid19-pdf-8957587789]), an independent follow-up statistical analysis supported the findings of the study with respect to the results of cholecalciferol treatment (160). Another trial of 986 patients hospitalized for COVID-19 in three UK hospitals in which cholecalciferol (≥280,000 IU in a time period of 7 weeks) was administered to 151 patients found an association with a reduced risk of COVID-19 mortality, regardless of baseline 25-hydroxyvitamin D levels (161). However, a double-blind, randomized, placebo-controlled trial of 240 hospitalized COVID-19 patients in São Paulo, Brazil, administered a single 200,000 IU oral dose of vitamin D. At the end of the study, there was a 24 ng/ml difference in 25-hydroxyvitamin D levels between the treatment group and the placebo group (*P = *0.001), and 87% of the members of the treatment group were vitamin D sufficient versus ∼11% in the placebo group. Supplementation was well tolerated. However, there was no reduction in the length of hospital stay or mortality, and no change to any other relevant secondary outcomes was reported (162). These early findings are thus still inconclusive with regard to the therapeutic value of vitamin D supplementation. However, other trials are under way, including one trial that is investigating the utility of vitamin D as an immune-modulating agent by monitoring whether administration of vitamin D precipitates an improvement of health status in nonsevere symptomatic COVID-19 patients and whether vitamin D prevents patient deterioration (163). Other trials are examining various factors, including mortality, symptom recovery, severity of disease, rates of ventilation, inflammatory markers, such as C-reactive protein and IL-6, blood cell counts, and the prophylactic capacity of vitamin D administration ([Bibr B163][Bibr B164][Bibr B166]). Concomitant administration of vitamin D with pharmaceuticals, such as aspirin (167), and bioactive molecules, such as resveratrol (168), is also under investigation.

The effectiveness of vitamin D supplementation against COVID-19 remains open for debate. All the same, there is no doubt that vitamin D deficiency is a widespread issue and should be addressed not only because of its potential link to SARS-CoV-2 incidence (169) but also because of its importance for overall health. There is a possibility that safe exposure to sunlight improves the endogenous synthesis of vitamin D, potentially strengthening the immune system. However, sun exposure is not sufficient on its own, particularly in the winter months. Indeed, while the possible link between vitamin D status and COVID-19 is further investigated, preemptive supplementation of vitamin D and encouraging people to maintain a healthy diet for optimum vitamin D status is likely to raise serum levels of 25-hydroxyvitamin D while being unlikely to carry major health risks. These principles seem to be the basis of a number of guidelines issued by some countries and scientific organizations that have advised supplementation of vitamin D during the pandemic. The Académie Nationale de Médecine in France recommends rapid testing of 25-hydroxyvitamin D for people over 60 years of age to identify those most at risk of vitamin D deficiency and advises them to obtain a bolus dose of 50,000 to 100,000 IU vitamin D to limit respiratory complications. It has also recommended that those under 60 years old should take 800 to 1,000 IU daily if they receive a SARS-CoV-2-positive test (170). In Slovenia, doctors have been advised to provide nursing home patients with vitamin D (171). Both Public Health England and Public Health Scotland have advised members of the Black, Asian, and minority ethnic communities to supplement vitamin D in light of evidence that they may be at higher risk for vitamin D deficiency along with other COVID-19 risk factors, a trend that has also been observed in the United States (172, 173). However, other UK scientific bodies, including the NICE, recommend that individuals supplement vitamin D as per usual UK government advice but warn that people should not supplement vitamin D solely to prevent COVID-19. All the same, the NICE has provided guidelines for research to investigate the supplementation of vitamin D in the context of COVID-19 (174). Despite vitamin D deficiency being a widespread issue in the United States (175), the National Institutes of Health have stated that there is “insufficient data to recommend either for or against the use of vitamin D for the prevention or treatment of COVID-19” (176). These are just some examples of how public health guidance has responded to the emerging evidence regarding vitamin D and COVID-19. Outside of official recommendations, there is also evidence that individuals may be paying increased attention to their vitamin D levels, as a survey of Polish consumers showed that 56% of respondents used vitamin D during the pandemic (177). However, some companies have used the emerging evidence surrounding vitamin D to sell products that claim to prevent and treat COVID-19, which in one instance required a federal court to intervene and issue an injunction barring the sale of vitamin D-related products due to the lack of clinical data supporting these claims (178). It is clear that further studies and clinical trials are required to conclusively determine the prophylactic and therapeutic potential of vitamin D supplementation against COVID-19. Until such time that sufficient evidence emerges, individuals should follow their national guidelines surrounding vitamin D intake to achieve vitamin D sufficiency.

## PROBIOTICS

Probiotics are “live microorganisms that, when administered in adequate amounts, confer a health benefit on the host” (179). Some studies suggest that probiotics are beneficial against common viral infections, and there is modest evidence to suggest that they can modulate the immune response (180, 181). As a result, it has been hypothesized that probiotics may have therapeutic value worthy of investigation against SARS-CoV-2 (182). Probiotics and next-generation probiotics, which are more akin to pharmacological-grade supplements, have been associated with multiple potential beneficial effects for allergies, digestive tract disorders, and even metabolic diseases through their anti-inflammatory and immunomodulatory effects (183, 184). However, the mechanisms by which probiotics affect these various conditions likely differ among strains, with the ultimate effect of the probiotic depending on the heterogeneous set of bacteria present (184). Some of the beneficial effects of probiotics include reducing inflammation by promoting the expression of anti-inflammatory mediators, inhibiting Toll-like receptors 2 and 4, competing directly with pathogens, synthesizing antimicrobial substances or other metabolites, improving intestinal barrier function, and/or favorably altering the gut microbiota and the brain-gut axis (184–186). It is also thought that lactobacilli such as Lactobacillus paracasei, Lactobacillus plantarum, and Lactobacillus rhamnosus have the capacity to bind to and inactivate some viruses via adsorptive and/or trapping mechanisms (187). Other probiotic lactobacilli and even nonviable bacterium-like particles have been shown to reduce both viral attachment to host cells and viral titers, along with reducing cytokine synthesis, enhancing the antiviral alpha interferon (IFN-α) response, and inducing various other antiviral mechanisms (187–195). These antiviral and immunobiotic mechanisms and others have been reviewed in detail elsewhere (32, 182, 196). However, there is also a bidirectional relationship between the lungs and gut microbiota known as the gut-lung axis (197), in which gut microbial metabolites and endotoxins may affect the lungs via the circulatory system and the lung microbiota in return may affect the gut (198). Therefore, the gut-lung axis may play a role in our future understanding of COVID-19 pathogenesis and become a target for probiotic treatments (199). Moreover, as microbial dysbiosis of the respiratory tract and gut may play a role in some viral infections, it has been suggested that SARS-CoV-2 may interact with our commensal microbiota (32, 200) and that the lung microbiome may play a role in developing immunity to viral infections (201). These postulations, if correct, might lead to the development of novel probiotic and prebiotic treatments. However, significant research is required to confirm these associations and their relevance to patient care, if any.

Probiotic therapies and prophylactics may also confer some advantages for managing symptoms of COVID-19 or risks associated with its treatment. Probiotics have tentatively been associated with the reduction of risk and duration of viral upper respiratory tract infections (202–204). Some meta-analyses that have assessed the efficacy of probiotics in viral respiratory infections have reported moderate reductions in the incidence and duration of infection (203, 205). Indeed, randomized controlled trials have shown that administering Bacillus subtilis and Enterococcus faecalis (206), Lactobacillus rhamnosus GG (207), or Lactobacillus casei and Bifidobacterium breve with galactooligosaccharides (208) via a nasogastric tube to ventilated patients reduced the occurrence of ventilator-associated pneumonia in comparison to that in the respective control groups in studies of viral infections and sepsis. These findings were also supported by a recent meta-analysis (209). Additionally, COVID-19 patients carry a significant risk of ventilator-associated bacterial pneumonia (210), but it can be challenging for clinicians to diagnose this infection due to the fact that severe COVID-19 infection presents with the symptoms of pneumonia (211). Therefore, an effective prophylactic therapy for ventilator-associated pneumonia in severe COVID-19 patients would carry significant therapeutic value. Additionally, in recent years, probiotics have become almost synonymous with the treatment of gastrointestinal issues due to their supposed anti-inflammatory and immunomodulatory effects (212). Notably, gastrointestinal symptoms commonly occur in COVID-19 patients (213), and angiotensin-converting enzyme 2, the portal by which SARS-CoV-2 enters human cells, is highly expressed in enterocytes of the ileum and colon, suggesting that these organs may be a potential route of infection (214, 215). Indeed, SARS-CoV-2 viral RNA has been detected in human feces (216, 217), and fecal-oral transmission of the virus has not yet been ruled out (218). Rectal swabs of some SARS-CoV-2-positive pediatric patients persistently tested positive for several days despite negative nasopharyngeal tests, indicating the potential for fecal viral shedding (219). However, there is conflicting evidence for the therapeutic value of various probiotics against the incidence or severity of gastrointestinal symptoms in viral or bacterial infections, such as gastroenteritis (220, 221). Nevertheless, it has been proposed that the administration of probiotics to COVID-19 patients and health care workers may prevent or ameliorate the gastrointestinal symptoms of COVID-19, a hypothesis that several clinical trials are now preparing to investigate (222, 223). Other studies are investigating whether probiotics may affect patient outcomes following SARS-CoV-2 infection (224).

Generally, the efficacy of probiotic use is a controversial topic among scientists. In Europe, EFSA has banned the term probiotics on products labels, which has elicited either criticism for EFSA or support for probiotics from researchers in the field (179, 225, 226). This regulation is due to the hyperbolic claims placed on the labels of various probiotic products, which lack rigorous scientific data to support their efficacy. Overall, the data supporting probiotics in the treatment or prevention of many different disorders and diseases is not conclusive, as the quality of the evidence is generally considered low (202). However, in the case of probiotics and respiratory infections, the evidence seems to be supportive of their potential therapeutic value. Consequently, several investigations are under way to investigate the prophylactic and therapeutic potential of probiotics for COVID-19. The blind use of conventional probiotics for COVID-19 is currently cautioned against until the pathogenesis of SARS-CoV-2 can be further established (227). Until clinical trials investigating the prophylactic and therapeutic potential of probiotics for COVID-19 are complete, it is not possible to provide an evidence-based recommendation for their use. Despite these concerns, complementary use of probiotics as an adjuvant therapeutic has been proposed by the Chinese National Health Commission and National Administration of Traditional Chinese Medicine (228). While supply issues prevented the probiotics market from showing the same rapid response to COVID-19 as some other supplements, many suppliers are reporting growth during the pandemic (229). Therefore, the public response once again seems to have adopted supplements promoted as bolstering the immune response despite a lack of evidence suggesting that they are beneficial for preventing or mitigating COVID-19.

## DISCUSSION

In this review, we report the findings to date of analyses of several dietary supplements and nutraceuticals. While existing evidence suggests potential benefits of n-3 PUFA and probiotic supplementation for COVID-19 treatment and prophylaxis, clinical data are still lacking, although trials are under way. Both zinc and vitamin C supplementation in hospitalized patients seem to be associated with positive outcomes; however, further clinical trials are required. In any case, vitamin C and zinc intake are part of a healthy diet and likely present minimal risk when used as supplements, though their potential prophylactic or therapeutic effects against COVID-19 are yet to be determined. On the other hand, mounting evidence from observational studies indicates that there is an association between vitamin D deficiency and COVID-19 incidence, and this association has also been supported by meta-analysis (230). Indeed, scientists are working to confirm these findings and to determine whether a patient’s serum 25-hydroxyvitamin D levels are also associated with COVID-19 severity. Clinical trials are required to determine whether preemptive vitamin D supplementation may mitigate against severe COVID-19. In terms of the therapeutic potential of vitamin D, initial evidence from clinical trials is conflicting but seems to indicate that vitamin D supplementation may reduce COVID-19 severity (159). The various clinical trials under way will be imperative to provide information on the efficacious use of vitamin D supplementation for COVID-19 prevention and/or treatment.

The purported prophylactic and therapeutic benefits of dietary supplements and nutraceuticals for multiple disorders, diseases, and infections has been the subject of significant research and debate for the last few decades. Inevitably, scientists are also investigating the potential for these various products to treat or prevent COVID-19. This interest also extends to consumers, which led to a remarkable increase of sales of dietary supplements and nutraceuticals throughout the pandemic due to a desire to obtain additional protections from infection and disease. The nutraceuticals discussed in this review, namely, vitamin C, vitamin D, n-3 PUFA, zinc, and probiotics, were selected because of potential biological mechanisms that may beneficially affect viral and respiratory infections and because they are currently under clinical investigation. Specifically, these compounds have all been found to influence cellular processes related to inflammation. Inflammation is particularly relevant to COVID-19 because of the negative outcomes (often death) observed in a large number of patients whose immune response becomes hyperactive in response to SARS-CoV-2, leading to severe outcomes, such as ARDS and sepsis (231). Additionally, there is a well-established link between diet and inflammation (232), potentially mediated in part by the microbiome (233). Thus, the idea that dietary modifications or supplementation might be used to modify the inflammatory response is tied to a broader view of how diet and the immune system are interconnected. The supplements and nutraceuticals discussed here therefore lie in sharp contrast to other alleged nutraceutical or dietary supplements that have attracted attention during the pandemic, such as colloidal silver (234), that have no known nutritional function and can be harmful. Importantly, while little clinical evidence is available about the effects of any supplements against COVID-19, the risks associated with those discussed above are likely to be low, and in some cases, they can be obtained from dietary sources alone.

There are various other products and molecules that have garnered scientific interest and might merit further investigation. These include polyphenols, lipid extracts, and tomato-based nutraceuticals, all of which have been suggested for the potential prevention of cardiovascular complications of COVID-19, such as thrombosis (32, 69). Melatonin is another supplement that has been identified as a potential antiviral agent against SARS-CoV-2 by computational methods (235), and it has also been highlighted as a potential therapeutic agent for COVID-19 due to its documented antioxidant, antiapoptotic, immunomodulatory, and anti-inflammatory effects (69, 236, 237). Notably, melatonin, vitamin D, and zinc have attracted public attention because they were included in the treatment plan of the former president of the United States upon his hospitalization due to COVID-19 (238). These are just some of the many substances and supplements that are under investigation but as of yet lack evidence to support their use for the prevention or treatment of COVID-19. While there is plenty of skepticism put forward by physicians and scientists surrounding the use of supplements, these statements have not stopped consumers from purchasing these products, with one study reporting that online searches for dietary supplements in Poland began trending with the start of the pandemic (177). Additionally, supplement usage increased between the first and second waves of the pandemic. Participants reported various reasons for their use of supplements, including to improve immunity (60%), to improve overall health (57%), and to fill nutrient gaps in their diet (53%). Other efforts to collect large data sets regarding such behavior have also sought to explore a possible association between vitamin or supplement consumption and COVID-19. An observational analysis of survey responses from 327,720 users of the COVID Symptom Study App found that the consumption of n-3 PUFA supplements, probiotics, multivitamins, and vitamin D was associated with a lower risk of SARS-CoV-2 infection in women but not in men after adjustment for potential confounders (239). According to the authors, the sexual dimorphism observed may in part be because supplements may better support females due to known differences between the male and female immune systems, or it may be due to behavioral and health consciousness differences between the sexes (239). Certainly, randomized controlled trials are required to investigate these findings further.

Finally, it is known that a patient’s nutritional status affects health outcomes in various infectious diseases (5), and COVID-19 is no different (3, 240, 241). Some of the main risk factors for severe COVID-19, which also happen to be linked to poor nutritional status, include obesity, hypertension, cardiovascular diseases, type II diabetes mellitus, and indeed age-related malnutrition (1, 3, 242). Although not the main focus of this review, it is important to consider the nutritional challenges associated with severe COVID-19 patients. Hospitalized COVID-19 patients tend to report an unusually high loss of appetite preceding admission, some suffer diarrhea and gastrointestinal symptoms that result in significantly lower food intake, and patients with poorer nutritional status are more likely to have worse outcomes and require nutrition therapy (243). Dysphagia also seems to be a significant problem in pediatric patients that suffer multisystem inflammatory syndrome (244) and rehabilitating COVID-19 patients, potentially contributing to poor nutritional status (245). Almost two-thirds of discharged COVID-19 ICU patients exhibit significant weight loss, of which 26% had weight loss greater than 10% (241). As investigated in this review, hospitalized patients also tend to exhibit vitamin D deficiency or insufficiency, which may be associated with greater disease severity (230). Therefore, further research is required to determine how dietary supplements and nutraceuticals may contribute to the treatment of severely ill and rehabilitating patients, who often rely on enteral nutrition.

## CONCLUSIONS

Despite all the potential benefits of nutraceutical and dietary supplement interventions presented, currently there is a paucity of clinical evidence to support their use for the prevention or mitigation of COVID-19 infection. Nevertheless, optimal nutritional status can prime an individual’s immune system to protect against the effects of acute respiratory viral infections by supporting normal maintenance of the immune system (1, 5). Nutritional strategies can also play a role in the treatment of hospitalized patients, as malnutrition is a risk to COVID-19 patients (245). Overall, supplementation of vitamin C, vitamin D, and zinc may be an effective method of ensuring their adequate intake to maintain optimal immune function, which may also convey beneficial effects against viral infections due to their immunomodulatory effects. Individuals should pay attention to their nutritional status, particularly their intake of vitamin D, considering that vitamin D deficiency is widespread. The prevailing evidence seems to indicate an association between vitamin D deficiency and COVID-19 incidence and, potentially, severity (134). As a result, some international authorities have advised the general public, particularly those at high risk of infection, to consider vitamin D supplementation. However, further well-controlled clinical trials are required to confirm these observations.

Many supplements and nutraceuticals designed for various ailments that are available in the United States and beyond are not strictly regulated (246). Consequently, there can be safety and efficacy concerns associated with many of these products. Often, the vulnerable members of society can be exploited in this regard, and unfortunately, the COVID-19 pandemic has proven no different. As mentioned above, the FDA has issued warnings to several companies for advertising falsified claims in relation to the preventative and therapeutic abilities of their products against COVID-19 (247). Further intensive investigation is required to establish the effects of these nutraceuticals, if any, against COVID-19. Until more effective therapeutics are established and vaccines have been widely distributed, it is important that public health agencies encourage the donning of face masks, promote physical distancing, and encourage good hygiene practices such as hand washing with soap (https://www.cdc.gov/coronavirus/2019-ncov/prevent-getting-sick/prevention.html), along with continuing expansive testing and contact tracing to mitigate SARS-CoV-2 spread (248, 249). Indeed, in light of this review, it would also be pertinent to adopt a healthy diet and lifestyle following national guidelines in order to maintain optimal immune health. Because of the broad public appeal of dietary supplements and nutraceuticals, it is important to evaluate the evidence regarding the use of such products. We will continue to update this review as more findings become available.

## Supplementary Material

Reviewer comments
