# Supplementary material for: Dietary Supplements and Nutraceuticals under Investigation for COVID-19 Prevention and Treatment
Source: mSystems. 2021 May 4;6(3):e00122-21. doi: 10.1128/mSystems.00122-21 (PMC8269209; doi:10.1128/mSystems.00122-21)
Supplement: Reviewer comments [file reviewer-comments.pdf]

AMERICAN SOCIETY FOR MICROBIOLOGY | mSystems®

Comprehensive scanning of prophages in *Lactobacillus*: distribution, diversity, antibiotic resistance genes, and linkages with CRISPR-Cas systems

Zhangming Pei, Faizan Sadiq, Xiao Han, Jianxin Zhao, Hao Zhang, R. Paul Ross, Wenwei Lu, and Wei Chen

Corresponding Author(s): Wei Chen, Jiangnan University

Review Timeline:

Submission Date:	November 17, 2020
Editorial Decision:	December 28, 2020
Revision Received:	February 23, 2021
Accepted:	May 11, 2021

Editor: Lee Ann McCue

Reviewer(s): Disclosure of reviewer identity is with reference to reviewer comments included in decision letter(s). The following individuals involved in review of your submission have agreed to reveal their identity: Dele Ogunremi (Reviewer #1)

Transaction Report:

(Note: With the exception of the correction of typographical or spelling errors that could be a source of ambiguity, letters and reports are not edited. The original formatting of letters and referee reports may not be reflected in this compilation.)

DOI: https://doi.org/10.1128/mSystems.01211-20

December 28, 2020

Prof. Wei Chen
Jiangnan University
1800 Lihu Ave, Wuxi, Jiangsu 214122, P.R. China.
Wuxi, Jiangsu 0510
China

Re: mSystems01211-20 (Comprehensive scanning and characterization of prophages in *Lactobacillus* reveals an uneven distribution and a potential correlation with the CRISPR-Cas system)

Dear Prof. Wei Chen:

Thank you for submitting your manuscript for publication in mSystems. It was reviewed by three reviewers whose assessments are attached. The reviewers agree that the study is of interest to mSystems readers, describing a substantive contribution to the field with key data and analyses toward understanding prophage in lactobacilli. However, the reviewers identified a number of items requiring revision/clarification. The points raised by the reviewers are important and merit your consideration. Please take careful note of the reviewers concerns related to interpretation of your results and presentation of conclusions. I hope that you will be able to make the changes needed to satisfy the requests of the reviewers, or respond convincingly to rebut them. I would be pleased to receive a revised version of your manuscript in which the necessary alterations have been made.

Below you will find the comments of reviewer#3, provided in addition to the attachments.

To submit your modified manuscript, log onto the eJP submission site at https://msystems.msubmit.net/cgi-bin/main.plex. If you cannot remember your password, click the "Can't remember your password?" link and follow the instructions on the screen. Go to Author Tasks and click the appropriate manuscript title to begin the resubmission process. The information that you entered when you first submitted the paper will be displayed. Please update the information as necessary. Provide (1) point-by-point responses to the issues raised by the reviewers as file type "Response to Reviewers," not in your cover letter, and (2) a PDF file that indicates the changes from the original submission (by highlighting or underlining the changes) as file type "Marked Up Manuscript - For Review Only."

Due to the SARS-CoV-2 pandemic, our typical 60 day deadline for revisions will not be applied. I hope that you will be able to submit a revised manuscript soon, but want to reassure you that the journal will be flexible in terms of timing, particularly if experimental revisions are needed. When you are ready to resubmit, please know that our staff and Editors are working remotely and handling submissions without delay. If you do not wish to modify the manuscript and prefer to submit it to another journal, please notify me of your decision immediately so that the manuscript may be formally withdrawn from consideration by mSystems.

If your manuscript is accepted for publication, you will be contacted separately about payment when the proofs are issued; please follow the instructions in that e-mail. Arrangements for payment

must be made before your article is published. For a complete list of **Publication Fees**, including supplemental material costs, please visit our website.

Corresponding authors may join or renew ASM membership to obtain discounts on publication fees. Need to upgrade your membership level? Please contact Customer Service at Service@asmusa.org.

Thank you for submitting your paper to mSystems.

Sincerely,

Lee Ann McCue

Editor, mSystems

Journals Department
American Society for Microbiology
1752 N St., NW
Washington, DC 20036
E-mail: peerreview@asmusa.org
Phone: 1-202-942-9338

Reviewer comments:

Reviewer #3 (Comments for the Author):

Pei and his colleagues have screened for prophage sequences in over 1000 Lactobacillus genomes, using software PHASTER. In this paper, the authors reported and characterized the abundant presence of predicted prophage regions in Lactobacillus genomes. They detected certain antibiotic resistance genes in the prophage sequences (such as ciprofloxacin resistance in Lactobacillus plantarum). They also attempted to correlate the distribution of CRISPR-Cas systems with prophage in Lactobacillus genomes. They reported a possible antagonistic relationship between CRISPR type I/II but not type [illegible] and the prevalence of intact prophages.
It has been known that prophages are highly prevalent in Lactobacillus genomes. However, very little is known regarding its function and relationship with its host. Multiple methods exist to look for prophages in bacterial genomes (such as PHASTER used in this paper). However, most of these tools generate results that can be inconclusive. With that being said, the authors in this paper filtered out the "incomplete or questionable" prophage prediction which was a good practice to clean up the dataset. To my knowledge, this is the first paper that has screened such a large number of Lactobacillus genomes to characterize prophage distribution. However, when dealing with a large number of genomes, certain granularity can be lost. I think the authors can dig a bit more on the correlation between prophage and CRISPR. For example, match between spacers and prophage sequence, prophage-mediated anti-CRISPR and etc. Regarding the analyses in this paper, please refer to the manuscript for specific comments

Pei et al.: Comprehensive scanning and characterization of prophages in *Lactobacillus* reveals an uneven distribution and a potential correlation with CRISPR-Cas system.

General

This paper is an in-depth look at the prophages in the genus *Lactobacillus* and could represent a very substantial contribution to the field of prophage biology and antimicrobial resistance after authors have addressed some of the items identified below.

The presence of antimicrobial resistance genes (ARG) in *Lactobacillus* is well known (see Asiminova and Yarullina, 2019 *Current Microbiology* 76:1407). In addition, the presence of antibiotic resistance genes (ARGs) in prophages have previously been reported in bacteria (e.g., Colavecchio et al., 2017 *Frontiers in Microbiology* 8:1108). This paper, however, uniquely reported a large number of ARGs in *Lactobacillus.* Thus, the verification of these ARGs in this genus will be an important contribution to the literature. Although authors reported phenotypic data based on MIC values, these results were not well presented. The illustrations should include the standard cut-off point for each antibiotic and inferences on susceptibility or resistance (see Table S5 under Specific comments). Importantly, the degree of agreement between the genotypic and phenotypic results, even if only for the 4 antibiotics tested, should be comprehensively described with the goal of providing a validation for all the genes detected. If the agreement is good, it should not be necessary to carry out further MIC testing because the proof of concept would have been established.

The word "correlation" in scientific writing requires a quantitative description and especially an estimation of r and p value. Because such quantitative description has not been made in this study, I will recommend a change in title: Comprehensive scanning and ………….uneven distribution and evaluation of a role for the CRISPR-Cas system.

The arguments for the inverse relationship between prophages and CRISPR-Cas is rather suspect. It is difficult to justify excluding a substantial amount of your data (7 out of 16 strains) and base a general conclusion on the remaining data. In this case, it appears there is evidence for and against the conclusion of the authors. This aspect of the paper needs to be re-written and authors should go where the evidence leads. If the data do not provide a strong evidence of an inverse relationship between the numbers of prophages and CRISPR-Cas, so be it!

There is a circular and an unproductive argument about the role of the different habitats in determining the number of prophages in *Lactobacillus* spp. (Line 120 – 139). The authors presented the hypothesis. Soon enough the data presented is not supporting the hypothesis, and rather than discard the hypothesis, there is an attempt to explain the "discrepancy" as if once that information is available, the hypothesis can then be proven. But this is a false logic. Evidence generated in this course of a study like

this one either supports a hypothesis or they do not. There is no need to be explaining why there is a discrepancy if the hypothesis is not holding. In that event, the alternate hypothesis needs to be looked at more favourably.

Specific

Abstracts needs a bit more work to make it clear.

Line 25-26 …………… about the mechanism of action of *Lactobacillus* prophages in regulating bacterial populations in the gut given the lack of information about prophage distribution, the complex genetic architecture, and uncertain relationships with their hosts.

Line 29: Remove: existing

Line 30-31 This sentence is not clear. Is it the objective of this paper to expand the datasets of prophage genomes? If yes, state so. Then you can indicate that the effort led to the finding of an uneven prophage distribution, and so on.

Line 31 dominated by different species? Not sure if "dominated" is the word to use here.

Line 32 The phrase "highly variable prophage genome diversity" reads like a tautology - "highly variable" and "diversity" (e.g., in a population) is saying the same thing twice in the same breath.

Rephrase.

Line 43: Remove the duplicate "in"

Line 46: Cas system is important for the understanding the co-evolution……………

Line 47: Rephrase "the safety of their presence….." Intent is not clear.

Line 58: prophages do not only increase………………………..but can even convert……………

Line 80: Can abbreviate *Lactobacillus* as *L.*

Line 125 *ruminis,* which occupy a restricted habitat, appeared to carry……………..

Line 126-127: Rewrite sentence for clarity. It is a bit confusing. The two species have the same number of intact prophages but different number of (total?) prophages: not clear how the environment might be a factor?

Line 129 These results suggest that………….

This sentence is the authors' conclusion from their observations but soon enough this is contradicted, at least when it comes to intact prophages, in Line 136-138.

"multi-niches-derived" should be better described in regular prose e.g., species that tended to occupy multiple niches…..

Line131 To further investigate whether………by the strain is related to hits habitat, 51 *L. brevis* , 133………

Line 139 – 146 See below under Figure 1.

Line 149: State the deviation from the central tendency represented as ±. The values given should follow the non-parametric analysis used in the Legend of Figure 2 (e.g., Median ± Interquartile range).

Line 151: Do not state "significant differences" were observed without following up with the statistics and p value.

Line 153 Change "significant outliers" or provide the statistics.

Line 154: ……. Revealing a high variation in genome sizes among prophages within and between bacteria species.

Line 157: …. to assess the evolution of

Line 160-164 Why is the GC content of *Lactobacillus* species (hosts) so variable? Did they descend from different ancestors (i.e., polyphyl)?

Line 192-197 The argument here is not persuasive. Are these independent clusters or a single cluster? If a single cluster, should the organisms belong to the same species even though they are given different names now? What is “species richness”? If the taxa are independent, why are they sharing clusters?

Line 205: The title points to the diversity in prophages belonging to the different clusters, however, the description that follows deals with “combined clusters”.

Line 208: …………….in a single cluster deserved to be linked or be segregated….

Line 233 – 235: Rearrange sentence such that “respectively” is as close to the two strains as possible...

…………..62 ARGS on 42….

……..261 ARGs consisted of 36 different determinations including. Lincosamides

Line 239: …………aac(6’)-Ie-aph2-Ia

Line 245-246 …….19 strains had it on a questionable or an incomplete……………..

Line 257-263 The logic in this paragraph is not clear. Virulence genes were searched for in the prophage genomes and some were found to be involved in extracellular polysaccharides metabolism but these were dismissed as virulence genes because they participated in adsorption and colonization (only)?

Line 257, 258: …………………virulence factors…………..

Line 260: codes for genes involved in extracellular polysaccharides metabolism.

What does protein-related genes mean? All proteins are encoded for by genes.

Line 262-263 The last sentence is confusing. Previously (lines 258-260), authors find virulence genes in prophages. Yet the conclusion is that there are no virulence genes in prophages?

Line 274: Percentage of what?

See other below comments on Fig. 5.

Line 288: Change correlation to relationship.

Correlation is the description of an arithmetic/statistical attribute that requires support with adequate metrics namely, i.e., r or r^2 and a probability value, p.

Move the sentence (line 288-289), which is a conclusion to after line 302 or into Discussion (i.e., after the reader has seen the evidence or results which are provided in line 289-302.

I am not sure a scientific justification can be made for excluding 7 of the 16 strains and then making a conclusion about an inverse relationship (of prophages and CRISPR-Cas) in the 9 remaining strains. A strong and scientifically valid rationale will be required. This analysis should be subjected to a test of significance.

Line 297: The sentence indicate that there is no significant difference between the number of prophage in the two groups seems to argue against the inverse relationship between CRISPR and prophages.

Line 312-313: ………. IIA CRISPR-Cas defence do not efficiently restrict temperate phage……………

Line 315-316: ………… (Fig. 7D, 7E) suggesting that Type I/III………………elements against *Lactobacillus*….

Line 319: …..spacers did not correlate with the number of intact………………………..[provide r and p values].

Lines 328, 331 Remove /

Line 350: ………. behind the variation in prophage occurrence in their genomes.

Line 354: "activation of functional genes" is vague.

Line 359: …………….niche-specificity…………………….

Line 364: ………… observed fewer intact…………..

Line 366 - 367: *Lactobacillus* prophage display wide variations in their genome sizes and GC contents.

Line 403: their described???

Line 404: ………..much fewer CRISPR-Cas

Line 428: ………………… species and showed the……………

Line 433: *Lactobacillus* for agriculture and human nutritional applications.

Line 452 **Genome sequencing and draft assembly**

Line 453: Genome sequencing was…………………..

Line 465: Rewrite sentence starting with "Construct a square matrix….". Was only one matrix constructed? Why do you have triangles in a square? Remove any confusion in the description and us an active voice to describe what was done.

Line 472: Change "virulent" to "virulence"

Line 477: Justify use of ≥30% amino acid identity as threshold or cite a supporting paper.

Line 515 - 516: ………….Collaborative Innovation Center of Food Safety and Quality Control………..

Figures

Figure 1 (page 8)

Fig 1 C is actually a table and should not be presented as a figure. Once Fig 1 C is removed, then the legend of the figure fits better (For example, a table does not have a y-axis or x-axis). The title of the table should be clearly presented.

Since the median is shown in the table, providing another average is unnecessary instead a range of prophages will be more insightful.

Fig. 4 The words on top of the bars in each panel are written in too small a font. Enlarge.

Fig. 5A The fonts are too small including both provided on the x axis as well as the y axis – on both sides of the heatmap.

Fig. 5B This illustration is not very informative. It should be converted into a Table with headings for each column. Information in the first column should be better presented for clarity. The parenthesis in the first column (copy numbers) is open to misinterpretation when you have many different ARGs – how do you know which one has a single copy and which has multiple copies from the illustration provided?

Fig. 7 Change "correlations" to "associations"

See rationale above, i.e., comment regarding line 288.

Table S6 The antibiotic sensitivity data should include the thresholds for the each antibiotic and an inference on whether each organism was Sensitive, Intermediate or Resistant.

References 9, 13, 20, 23, 24, 27, 33, 47, 48, 56, 59,71,72 Use sentence case for the title of the papers (instead of the title case).

Reference 56: Check title for accuracy

Dear Dr. Lee Ann McCue and three reviewers:

Thank you for your letter and the reviewers' comments on our manuscript entitled "**Comprehensive scanning of prophages in *Lactobacillus*: distribution, diversity, antibiotic resistance genes, and linkages with CRISPR-Cas systems**" (**mSystems01211-20R1**). We sincerely thank the three reviewers for the constructive comments and suggestions, which turned out to be very helpful for revising and improving our manuscript. We have considered and incorporated every comment of the reviewers and your careful editorial work on the manuscript in our revised manuscript whenever we can. Revisions are highlighted in blue in the file "**Marked Up Manuscript**" which we would like to submit for your kind consideration.

There is, in addition, a small mistake in the original manuscript that should be clarified. In the process of revising the manuscript, we found that the three genomes obtained from the NCBI Genome database (NCTC1407, NCTC13720, and NCTC13721) are wrongly classified into *Lactobacillus acidophilus*. Thus, we removed these three wrongly classified genomes, and they have been replaced by three other correct *L. acidophilus* genomes (s-13, s-4, and P2) in the revised manuscript. Neither the interpretation nor the conclusion of this study is affected by this error. The corresponding statistical data, figures, and Supplemental materials have been corrected.

The following are itemized response lists for each point raised by reviewers.

Reviewer #1

General:

This paper is an in-depth look at the prophages in the genus *Lactobacillus* and could represent a very substantial contribution to the field of prophage biology and antimicrobial resistance after authors have addressed some of the items identified below.

Author Reply: Thank you very much for your positive comments, constructive comments, and suggestions which would help us in-depth to improve the quality of our manuscript. Here are our responses to every comment.

Comment #1: The presence of antimicrobial resistance genes (ARG) in *Lactobacillus* is well known (see Anisimova and Yarullina, 2019 Current Microbiology 76:1407). In addition, the presence of antibiotic resistance genes (ARGs) in prophages have previously been reported in

bacteria (e.g., Colavecchio et al., 2017 Frontiers in Microbiology 8:1108). This paper, however, uniquely reported a large number of ARGs in *Lactobacillus*. Thus, the verification of these ARGs in this genus will be an important contribution to the literature. Although authors reported phenotypic data based on MIC values, these results were not well presented. The illustrations should include the standard cut-off point for each antibiotic and inferences on susceptibility or resistance (see Table S5 under Specific comments). Importantly, the degree of agreement between the genotypic and phenotypic results, even if only for the 4 antibiotics tested, should be comprehensively described with the goal of providing a validation for all the genes detected. If the agreement is good, it should not be necessary to carry out further MIC testing because the proof of concept would have been established.

Author Reply #1: Thank you so much for your careful reading and professional comments. The phenotypic data based on MIC values were not well presented, it was an oversight on our part. In the revised manuscript, we have now included figures with MIC value distributions, please see Fig. 5B. The "Sensitive" or "Resistant" of lactobacilli to antibiotics have also been added in Supplemental Table S3B. In this manuscript, we discovered that the putative *lmrD-efrA* resistance gene clusters exist in every *L. plantarum* strain, and most of them were mediated by prophages. Therefore, the main purpose of our phenotype experiments is to explore whether the whole species of *L. plantarum* has high resistance to certain antibiotics. Of course, we have also described the agreement between the genotypic and phenotypic results among the involved strains. For the detailed description, please see Line 263-300.

Comment #2: The word "correlation" in scientific writing requires a quantitative description and especially an estimation of r and p value. Because such quantitative description has not been made in this study, I will recommend a change in title: Comprehensive scanning and …………uneven distribution and evaluation of a role for the CRISPR-Cas system.

Author Reply #2: Thank you for your valuable advice. As you said, we have not made any quantitative description. Thus, we cannot use the word "correlation". To better show the kernel of this study, after careful consideration, we have made a further slight change to the recommended title, the final title is "Comprehensive scanning of prophages in *Lactobacillus*: distribution, diversity, antibiotic resistance genes and linkages with CRISPR-Cas systems". Once again, thank you very much for your constructive comment on our manuscript.

Comment #3: The arguments for the inverse relationship between prophages and CRISPR-Cas is rather suspect. It is difficult to justify excluding a substantial amount of your data (7 out of 16

strains) and base a general conclusion on the remaining data. In this case, it appears there is evidence for and against the conclusion of the authors. This aspect of the paper needs to be re-written and authors should go where the evidence leads. If the data do not provide a strong evidence of an inverse relationship between the numbers of prophages and CRISPR-Cas, so be it!

Author Reply #3: Thank you very much for your suggestion. As you said, although we found an inverse relationship between the numbers of prophages and CRISPR-Cas systems in 7 species (we performed significance analyses), it cannot be taken as a general conclusion. Thus, we deleted the part with low-level evidence and re-analyzed it. In the revised manuscript, the inverse relationships in some species were stated as an interesting finding. And we only reserved the part of the comparison of the number of intact prophages in *Lactobacillus* genomes with or without the CRISPR-Cas system, please see Line 311-331, Fig. 6B and 6C. In addition, for advancing our understanding of phage population diversity and bacteria-phage interactions, according to the Commented [A8] raised by Reviewer #3, we added a whole section of Results to introduce our new findings on the association between CRISPR spacers and prophages, including CRISPR spacer clustering, spacer-prophage alignment, and self-targeting statistics, please see Line 333-363, Fig. 7A, 7B, 7C, and 7D.

Comment #4: There is a circular and an unproductive argument about the role of the different habitats in determining the number of prophages in *Lactobacillus* spp. (Line 120 – 139). The authors presented the hypothesis. Soon enough the data presented is not supporting the hypothesis, and rather than discard the hypothesis, there is an attempt to explain the "discrepancy" as if once that information is available, the hypothesis can then be proven. But this is a false logic. Evidence generated in this course of a study like this one either supports a hypothesis or they do not. There is no need to be explaining why there is a discrepancy if the hypothesis is not holding. In that event, the alternate hypothesis needs to be looked at more favorably.

Author Reply #4: Thank you very much for your guidance! It was our thoughtlessness about this section. We have attempted to look for a relationship between the prophage and origin within a particular species, subsequently reported no such linkage was found. As you said, the logic of trying to explain this phenomenon is wrong, let alone for this hypothesis with low evidence. Therefore, we referred to the comment of Reviewer #3, we have investigated the relationship that exists among all *Lactobacillus* strains with predicted intact prophages instead of focusing on a particular species. Strains simply divided into the "human/mammal" group (*n* = 1022) and "fermented food" group (*n* =266), and an interesting result was found, strains from fermented food tended to harbor a significantly higher number of prophages than strains from human/mammal, please see Line 137-144, Fig. 1C and 1D. Furthermore,

we also put forward hypotheses and inferences about why lactobacilli from fermented food carry more prophages, please see Line 392-403.

Specific comments

Comment #5: Abstracts needs a bit more work to make it clear.

Author Reply #5: Thank you for your advice. We have taken your suggestions and performed a substantial rewrite of the Abstract and Importance, please see Line 23-51.

Comment #6: Line 25-26 …………… about the mechanism of action of *Lactobacillus* prophages in regulating bacterial populations in the gut given the lack of information about prophage distribution, the complex genetic architecture, and uncertain relationships with their hosts.

Author Reply #6: Thank you for your revision, we have taken your suggestion and made a slight change, please see Line 25-26.

Comment #7: Line 29: Remove: existing

Author Reply #7: Thank you for your revision, we have deleted the word "existing".

Comment #8: Line 30-31 This sentence is not clear. Is it the objective of this paper to expand the datasets of prophage genomes? If yes, state so. Then you can indicate that the effort led to the finding of an uneven prophage distribution, and so on.

Author Reply #8: Thank you very much for your suggestion. We have changed this sentence to "We present an uneven prophage distribution among *Lactobacillus* species, multi-habitat species retained more prophages in their genomes than restricted-habitat species", in Line 28-30.

Comment #9: Line 31 dominated by different species? Not sure if "dominated" is the word to use here.

Author Reply #9: Thank you for your suggestion. It is a confusing word, and we have been rephrased this sentence, please see Line 28-30.

Comment #10: Line 32 The phrase "highly variable prophage genome diversity" reads like a tautology - "highly variable" and "diversity" (e.g., in a population) is saying the same thing twice in the same breath. Rephrase.

Author Reply #10: Thank you for your advice. We have rephrased this sentence: "…

presented a high genome diversity of *Lactobacillus* prophages.", in Line 31-32.

Comment #11: Line 43: Remove the duplicate "in"

Author Reply #11: It was our oversight and mistake. Thanks for your revision. It has been deleted.

Comment #12: Line 46: Cas system is important for the understanding the co-evolution………..

Author Reply #12: Thank you for your revision, we have corrected this sentence in Line 50-51.

Comment #13: Line 47: Rephrase "the safety of their presence….." Intent is not clear.

Author Reply #13: Thank you very much for your suggestion. We have changed this sentence to "Our data of the prophage-encoded antibiotic resistance genes (ARGs) and the resistance phenotype of lactobacilli provide evidence for deciphering the putative role of prophages as vectors of the ARGs", in Line 47-49.

Comment #14: Line 58: prophages do not only increase………………………..but can even convert……………

Author Reply #14: Thank you for your revision, we have corrected it in Line 64-65.

Comment #15: Line 80: Can abbreviate *Lactobacillus* as *L.*

Author Reply #15: Thank you for your suggestion. However, refer to Comment #8 raised by Reviewer #2, for each species name that first appears in the text, we should use the full name. *Lactobacillus brevis*, *Lactobacillus ruminis*, and *Lactobacillus gasseri* are all mentioned in the text for the first time. Thus, in Line 87, the "*Lactobacillus*" should not be abbreviated as "*L.*".

Comment #16: Line 125 ruminis, which occupy a restricted habitat, appeared to carry……………...

Author Reply #16: Thank you for your revision, we have corrected it in Line 130-131.

Comment #17: Line 126-127: Rewrite sentence for clarity. It is a bit confusing. The two species have the same number of intact prophages but different number of (total?) prophages: not clear how the environment might be a factor?

Author Reply #17: Thank you for your suggestion. What we want to express here is the

detection rates (have intact prophage or not) of intact prophage in *L. paracasei* and *L. rhamnosus* are similar, but *L. paracasei* carried a higher number of intact prophages than *L. rhamnosus* (Refer to Table 1). *L. rhamnosus* is usually found in fermented foods, oral, and gastrointestinal tracts (Barrons & Tassone, 2008, *Clinical Therapeutics*), whereas *L. paracasei* can be isolated from a range of ecological niches (e.g., oral, gastrointestinal tract, feces, vagina, milk, cheese, dairy products, cereal products, dough, plants, and garbage) (Smokvina et al., 2013, *PLos one*). Therefore, based on those results, we suggest that multi-habitat species tend to retain more intact prophages. We recognized the sentences in Line 132-135.

Comment #18: Line 129 These results suggest that…………..

Author Reply #18: **Thank you for your revision, we have corrected it in Line 135.**

Comment #19: This sentence is the authors' conclusion from their observations but soon enough this is contradicted, at least when it comes to intact prophages, in Line 136-138. "multi-niches-derived" should be better described in regular prose e.g., species that tended to occupy multiple niches…..

Author Reply #19: **Thank you for your comments. We are sorry for the misunderstanding caused by the sentences in Line 136-138. What we want to express is that, in *L. plantarum* and *L. brevis*, the number of intact prophages carried by strains may not be related to its current isolation source. This finding does not conflict with other conclusions. Of course, refer to Comment #4, this is a false logic to explain the discrepancy. We have removed the whole part of this low credibility investigation and replaced it with another one (Refer to our reply on Comment #4). Moreover, we have corrected the sentence in Line 135-136: "…, species that tended to occupy multiple habitats…".**

Comment #20: Line131 To further investigate whether………by the strain is related to its habitat, 51 L. brevis , 133………

Author Reply #20: **Thank you for your revision, we have corrected it in Line 138.**

Comment #21: Line 139 – 146 See below under Figure 1.

Author Reply #21: **Thank you for your suggestion. Please refer to our reply on Comment #62.**

Comment #22: Line 149: State the deviation from the central tendency represented as ±. The

values given should follow the non-parametric analysis used in the Legend of Figure 2 (e.g., Median ± Interquartile range).

Author Reply #22: Thank you very much for your valuable suggestion. We have corrected it as (Median ± Interquartile range), please see Line 148.

Comment #23: Line 151: Do not state “significant differences” were observed without following up with the statistics and p value.

Author Reply #23: Thank you for your advice, we have added the *p*-value in Line 151.

Comment #24: Line 153 Change “significant outliers” or provide the statistics.

Author Reply #24: Thank you for your advice, we have deleted the word “significant” in Line 152.

Comment #25: Line 154: ……. revealing a high variation in genome sizes among prophages within and between bacteria species.

Author Reply #25: Thank you for your revision, we have corrected it in Line 153.

Comment #26: Line 157: …. to assess the evolution of

Author Reply #26: Thank you for your revision, we have corrected it in Line 155.

Comment #27: Line 160-164 Why is the GC content of *Lactobacillus* species (hosts) so variable? Did they descend from different ancestors (i.e., polyphyl)?

Author Reply #27: Thank you very much for your comments. We normally think that species of the *Lactobacillus* genus are descended from one common ancestor, (Sun et al., 2015, *Nat Commun*) have proven it through genus-wide phylogenetic tree based on core genes. According to the description of the *Lactobacillus* genus in Bergey's Manual of Systematic Bacteriology, the lactobacilli were grouped taxonomically according to their major carbohydrate metabolism, as homo-fermentative (Group A), facultatively heterofermentative (Group B) or obligately heterofermentative (Group C) lactobacilli. Obligately heterofermentative *Lactobacillus*, such as *L. fermentum*, *L. mucosae*, and *L. brevis*, the GC contents range from 45% to 51%; Facultatively heterofermentative *Lactobacillus*, such as *L. paracasei*, *L. rhamnosus*, and *L. plantarum*, the GC contents range from 44% to 47%; whereas for homo-fermentative *Lactobacillus*, such as *L. acidophilus*, *L. helveticus*, *L. gasseri*, *L. johnsonii*, and *L. salivarius*, the GC contents are generally low, range from 32% to 35%. Thus, the varied GC contents of *Lactobacillus* species are possibly due to a long

evolutionary process of this genus.

Comment #28: Line 192-197 The argument here is not persuasive. Are these independent clusters or a single cluster? If a single cluster, should the organisms belong to the same species even though they are given different names now? What is "species richness"? If the taxa are independent, why are they sharing clusters?

Author Reply #28: Thank you for your comments. We are sorry for these confusing sentences. In the original manuscript, due to incorrect clustering arrangement, it seems that this part in Line 192-197 is not convincing. Here, there should be no concept of "Combine cluster", it was our mistake. To avoid ambiguity and obscurity of expression, through the reanalysis and rewriting, we have made great changes to this part of the results. In revised Fig. 3, the 1,459 prophages were located in 11 independent clusters. That is, *L.gasseri*, *L. helveticus*, and *L. johnsonii* prophages belonged to one cluster; *L. fermentum* and *L. mucosae* prophages formed one cluster; *L. paracasei* and *L. rhamnosus* prophages constituted one cluster; the remaining eight clusters were composed of prophages from a single *Lactobacillus* species. Thus, we drew the following conclusion: in the *Lactobacillus* genus, the similarities between the prophages carried by species with the close genetic relationship are extremely high; and the farther the evolutionary relationship between host species, the lower the similarity between the prophages. Moreover, the "species richness" was also a confusing word, this sentence has also been rephrased into "owing to the presence of multiple relatively discrete species in the genus *Lactobacillus*, the whole *Lactobacillus* prophage population also reflects a considerable genetic diversity and numerous relatively independent taxa", for detailed description, please see Line 195-210 and Fig. 3.

Comment #29: Line 205: The title points to the diversity in prophages belonging to the different clusters, however, the description that follows deals with "combined clusters".

Author Reply #29: Thank you for your comments. As mentioned in our reply to Comment #28, the concept of "Combine cluster" was our fault. Therefore, according to the reanalysis results, we performed ANI analyses on independent clusters, and through the landscape visualization of representative prophages in every cluster, to present the extent of differences in the structures of each cluster/species, for changes in this part, please see Line 212-246, Fig. 4A-C, Supplemental Fig. S3A-C.

Comment #30: Line 208: ……………..in a single cluster deserved to be linked or be segregated….

Author Reply #30: **Thank you for your revision, we have corrected it in Line 215.**

Comment #31: Line 233 – 235: Rearrange sentence such that "respectively" is as close to the two strains as possible... …………..62 ARGS on 42…. ……..261 ARGs consisted of 36 different determinations including. Lincosamides

Author Reply #31: **Thank you for your advice. The sentences have been rearranged in Line 250-252.**

Comment #32: Line 239: …………aac(6')-Ie-aph2-Ia

Author Reply #32: **Thank you for your revision, we have corrected it in Line 256.**

Comment #33: Line 245-246 …….19 strains had it on a questionable or an incomplete………………

Author Reply #33: **Thank you for your revision, we have corrected it in Line 261.**

Comment #34: Line 257-263 The logic in this paragraph is not clear. Virulence genes were searched for in the prophage genomes and some were found to be involved in extracellular polysaccharides metabolism but these were dismissed as virulence genes because they participated in adsorption and colonization (only)?

Author Reply #34: **Thank you for your advice. Originally, we would like to investigate the distribution of virulence factors among *Lactobacillus* prophages after the description of ARGs. Although some potential virulence factors were identified, they belong to the genes that enhance bacterial host colonization. As we known, colonization genes of pathogenic bacteria are harmful to the host organism, whereas those of *Lactobacillus* are not virulence factors. Thus, we wrote those sentences in the original manuscript. However, to maintain the logical integrity of the revised manuscript, we decided to delete this part that may cause misunderstanding.**

Comment #35: Line 257, 258: ………………….virulence factors……………

Author Reply #35: **Thank you for your revision. This part that may cause misunderstanding has been deleted.**

Comment #36: Line 260: codes for genes involved in extracellular polysaccharides metabolism. What does protein-related genes mean? All proteins are encoded for by genes.

Author Reply #36: **We are sorry for this mistake. This part that may cause**

misunderstanding has been deleted.

Comment #37: Line 262-263 The last sentence is confusing. Previously (lines 258-260), authors find virulence genes in prophages. Yet the conclusion is that there are no virulence genes in prophages?

Author Reply #37: Thank you for your advice. Refer to our reply on Comment #34, this part has been deleted.

Comment #38: Line 274: Percentage of what? See other below comments on Fig. 5.

Author Reply #38: Thank you for your comment. As Comment #64, Fig. 5B hardly provides useful information. The section on antibiotic resistance has been reorganized, and this table has been deleted.

Comment #39: Line 288: Change correlation to relationship. Correlation is the description of an arithmetic/statistical attribute that requires support with adequate metrics namely, i.e., r or r2 and a probability value, p.

Author Reply #39: Thank you for your revision, we have reorganized and rewritten this section, please see Line 311-319.

Comment #40: Move the sentence (line 288-289), which is a conclusion to after line 302 or into Discussion (i.e., after the reader has seen the evidence or results which are provided in line 289-302.

Author Reply #40: Thank you for your advice. This sentence has been moved into the section "Discussion" Line 459-461.

Comment #41: I am not sure a scientific justification can be made for excluding 7 of the 16 strains and then making a conclusion about an inverse relationship (of prophages and CRISPR-Cas) in the 9 remaining strains. A strong and scientifically valid rationale will be required. This analysis should be subjected to a test of significance.

Author Reply #41: Thank you for your guidance. As you said before (Comment #3), it is unreliable that justify excluding a substantial amount of data (7 out of 16 strains) and base a general conclusion on the remaining data. Thus, we deleted the part with low level evidence and only reserved the part of comparison of the number of intact prophages in *Lactobacillus* genomes with or without CRISPR-Cas system.

Comment #42: Line 297: The sentence indicate that there is no significant difference between the number of prophage in the two groups seems to argue against the inverse relationship between CRISPR and prophages.

Author Reply #42: Thank you for your comment. We are sorry for these confused sentences. The original sentence was to explain there is no significant difference between the numbers of total predicted prophage fragments in CRISPR positive group and negative group, whereas the inverse relationships was occurs in predicted intact prophages. Refer to our reply on Comment #3, the investigations with low evidence and those confused sentences have been remove in the revised manuscript.

Comment #43: Line 312-313: ……… IIA CRISPR-Cas defense do not efficiently restrict temperate phage……………

Author Reply #43: Thank you for your revision, we have corrected it in Line 328.

Comment #44: Line 315-316: ………… (Fig. 7D, 7E) suggesting that Type I/III………………elements against Lactobacillus….

Author Reply #44: Thank you for your revision, we have corrected it in Line 330.

Comment #45: Line 319: …..spacers did not correlate with the number of intact………………………..[provide r and p values].

Author Reply #45: Thank you for your suggestion. This part has been reorganized and rewritten.

Comment #46: Lines 328, 331 Remove /

Author Reply #46: Thank you for your revision, we have deleted "/" in the legend of Fig. 6 (revised manuscript).

Comment #47: Line 350: ……… behind the variation in prophage occurrence in their genomes.

Author Reply #47: Thank you for your revision, we have corrected it in Line 377.

Comment #48: Line 354: "activation of functional genes" is vague.

Author Reply #48: Thank you for your advice. The sentence has been corrected in "by activating stress-responsive genes", please see Line 381.

Comment #49: Line 359: …………….niche-specificity…………………….

Author Reply #49: Thank you for your revision, we have corrected it in Line 386.

Comment #50: Line 364: …………. observed fewer intact…………..

Author Reply #50: Thank you for your revision, we have corrected it in Line 391.

Comment #51: Line 366 - 367: *Lactobacillus* prophage display wide variations in their genome sizes and GC contents.

Author Reply #51: Thank you for your revision, we have corrected it in Line 404.

Comment #52: Line 403: their described???

Author Reply #52: We are sorry for the mistake. It has been corrected in "…much higher than those described by Crawley et al.", please see Line 448.

Comment #53: Line 404: ……….much fewer CRISPR-Cas

Author Reply #53: Thank you for your revision, we have corrected it in Line 449.

Comment #54: Line 428: ………………… species and showed the……………

Author Reply #54: Thank you for your revision, we have corrected it in Line 508.

Comment #55: Line 433: *Lactobacillus* for agriculture and human nutritional applications.

Author Reply #55: Thank you for your revision, we have corrected it in Line 513.

Comment #56: Line 452 Genome sequencing and draft assembly

Author Reply #56: Thank you for your revision, we have corrected it in Line 531.

Comment #57: Line 453: Genome sequencing was…………………..

Author Reply #57: Thank you for your revision, we have corrected it in Line 538.

Comment #58: Line 465: Rewrite sentence starting with "Construct a square matrix….". Was only one matrix constructed? Why do you have triangles in a square? Remove any confusion in the description and us an active voice to describe what was done.

Author Reply #58: We are dreadfully sorry for this confusing description. Thank you for your suggestion, these sentences have been removed.

Comment #59: Line 472: Change “virulent” to “virulence”

Author Reply #59: Thanks for your revision. Refer to our reply on Comment #34, the relevant part has been deleted.

Comment #60: Line 477: Justify use of ≥30% amino acid identity as threshold or cite a supporting paper.

Author Reply #60: Thank you for your advice. We have added the reference (Ref. No 73: Campedelli et al., 2019, *Appl Environ Microbiol*) in Line 580.

Comment #61: Line 515 - 516: …………..Collaborative Innovation Center of Food Safety and Quality Control…………

Author Reply #61: Thanks for your revision. It has been corrected in Line 625-626.

Comment #62: Figure 1 (page 8)

Fig 1C is actually a table and should not be presented as a figure. Once Fig 1 C is removed, then the legend of the figure fits better (For example, a table does not have a y-axis or x-axis). The title of the table should be clearly presented.

Since the median is shown in the table, providing another average is unnecessary instead a range of prophages will be more insightful.

Author Reply #62: Thank you very much for your valuable suggestions. Fig. 1C has been removed and changed into Table 1. We have also provided the range of prophages in Table 1.

Comment #63: Fig. 4 The words on top of the bars in each panel are written in too small a font. Enlarge.

Author Reply #63: Thank you for your suggestion. Due to a large number of prophages, it cannot be enlarged to a clear size. The classification results of sub-clusters are cores of this figure. Thus, in order not to affect the readability of this figure, we put the name orders of every clusters into Supplemental Data Set Tab2.

Comment #63: Fig. 5A The fonts are too small including both provided on the x axis as well as the y axis – on both sides of the heatmap.

Author Reply #64: Thank you for your suggestion. The fonts on the x-axis have been enlarged. However for the y axis, due to a large number of prophages, it is impossible to enlarge to a clear size. And the specific prophage names do not affect the display of the conclusion, thus, we put the name order of the y axis of Fig. 5A into Supplemental Data Set

Tab3.

Comment #64: Fig. 5B This illustration is not very informative. It should be converted into a Table with headings for each column. Information in the first column should be better presented for clarity. The parenthesis in the first column (copy numbers) is open to misinterpretation when you have many different ARGs – how do you know which one has a single copy and which has multiple copies from the illustration provided?

Author Reply #65: Thank you for your advice. As you say, Fig. 5B hardly provides useful information. The section on antibiotic resistance has been reorganized, and this table has been deleted.

Comment #66: Fig. 7 Change "correlations" to "associations"

See rationale above, i.e., comment regarding line 288.

Author Reply #66: Thank you for your advice. It has been corrected in the legend of Fig. 6 (revised manuscript), please see in Line 962.

Comment #67: Table S6 The antibiotic sensitivity data should include the thresholds for the each antibiotic and an inference on whether each organism was Sensitive, Intermediate or Resistant.

Author Reply #67: Thank you for your suggestion. For five antibiotics with definite microbiological breakpoints, the "Sensitive" or "Resistant" for lactobacilli have been added in Supplemental Table S3B. The microbiological breakpoints of the other four antibiotics for lactobacilli have not yet been determined, we also made the corresponding description and inference in the revised manuscript, please see Line 278-289.

Comment #68: References 9, 13, 20, 23, 24, 27, 33, 47, 48, 56, 59, 71, 72 Use sentence case for the title of the papers (instead of the title case).

Author Reply #68: Thank you for your comment. These titles have been corrected.

Comment #69: Reference 56: Check title for accuracy

Author Reply #69: Thank you for your comment. It has been corrected.

Reviewer #2

General:

The authors provide a prophage prediction analysis on 1472 genomes from 16 different *Lactobacillus* species. They showed an uneven prophage distribution and a high diversity among these prophages. They highlighted antibiotic resistance genes and studied the distribution of CRISPR-Cas system across the genomes. The study of *Lactobacillus* prophages with such extended data is very interesting but clarifications need to be made.

Author Reply: Thank you very much for your positive comments. Your valuable advices and suggestions have greatly helped us to revise our manuscript. The following are our responses for each point.

Comment #1: The authors base the whole study on prophage regions predicted by PHASTER. The authors should state the limitations of using such a prediction tool for their analysis. When the authors refer to prophage of *Lactobacillus*, it should be clearly stated that these are "predicted" intact regions. Were predicted intact prophage regions verified for the presence of expected genes required for the production of a functional phage particle?

Author Reply #1: Thank you very much for your advice. We made a detailed statement about the shortcomings of using PHASTER to predict prophage, in the part of Discussion, Line 481-497. On the issue that should be clearly stated "predicted" intact prophage, thank you for your reminding, we have made corrections accordingly, such as "Abstract" Line 28; "Result" Line 123, Line 147, Line 165; "Discussion" Line 504; etc. All predicted intact prophages were previously verified for the presence of cornerstone genes through annotating with the NR databases. Based on the annotation results, we confirmed the existence of all five core modules' genes (Lysogeny, DNA replication, DNA packaging, Morphogenesis, and Lysis) in 81% (1,183/1,459) of the predicted intact *Lactobacillus* prophages in this study, 97% (1,420/1,459) of them have four or more core modules' genes. A small number of predicted intact prophages were annotated with a few known functional genes, but a large number of phage-like proteins. Therefore, we consider that most of these predicted intact prophages have relatively complete genomic organizations, and did not perform further simplification to the prediction results of PHASTER. As for whether these predicted intact prophages in this study can be induced and actually transformed on the bench, we can't confirm them one by one due to the different sensitivity of lactobacilli to MMC concentration (Oliveira et al., 2017. *Front Microbiol*). However, we have also performed verification in some strains, please refer to our reply on Comment #5.

Comment #2: Integration sites of the prophages could also be reported because this information is important to discuss the infectiousness and host specificity of the phages.

Author Reply #2: This is an excellent point! Thank you for your valuable suggestion. Refer to the methods of (Rezaei et al., 2019, *Nat Commun*) and (Brueggemann et al., 2017, *Sci Rep*), we have added the description and visualization on the integration sites of *Lactobacillus* prophages, please see section "Result" Line 170-183, section "Discussion" Line 417-423, and Fig. 2C.

Comment #3: The results on the diversity of *Lactobacillus* prophages among and within clusters is shallow (example lines 213-215) and not always clear to follow (usefulness of figure 4?). The authors report that some prophages are similar, maybe it would be interesting to compare these prophage and the genomes of their host strains and see what we can learn from these.

Author Reply #3: Thank you for your advice. To visually and intuitively demonstrate the extent of differences in the structures of *Lactobacillus* prophages for each cluster or species, the landscapes of representative intact prophages were depicted, please see Fig. 4C and Supplemental Fig. S3B. By combining with the landscape visualization of representative prophages and the clustering results of ANI analysis, we improved the usefulness of this part. For the detailed description, please see the section "Result" Line 228-246. Moreover, we have also tried to link these prophages to the genomes of their host strains, through constructing the phylogenetic tree of host bacteria based on the core genomes, however, we have not found any obvious connection yet. If you have any good idea for this aspect, please tell us, and we are very happy to use this batch of data to do an interesting investigation.

Comment #4: It would also be interesting to place the *Lactobacillus* prophages in a broader picture. How do they compare with other phages publicly available and with other LAB phages?

Author Reply #4: Thank you for your suggestion. We have compared all 1,459 putative intact prophage genomes with publicly available Lactobacillus phage sequences in GenBank, only 16.2% of them (236/1,459) matched with 29 published genomes, indicating that most of the intact prophages predicted in this study were probably new, the detailed results please see the section "Results" Line 162-169 and Supplemental Fig. S2. The insufficiency of viral sequence databases is a common problem faced by researchers, we expect more and more active phage genomes to be sequenced.

Comment #5: Unfortunately the authors do not say anything about the inducibility of potential prophage predicted among *Lactobacillus* genomes. It would have been interesting to correlate

dry/wet lab data (PHASTER predictions versus prophage inducibility)

Author Reply #5: Thank you very much for your comments. In another study (Pei et al., 2020, *Virus Res*), we previously selected 142 potential *Lactobacillus* 'lysogens' (which carry predicted intact prophages, and both involved in this study) from 6 different species to induce prophages using Mitomycin C (MMC). Several temperate phages were successfully induced and sequenced, through alignment, almost all of them matched the corresponding intact prophage regions predicted by PHASTER; however, the majority of strains did not respond to MMC induction (0.70μg/mL). That is because the sensitivities of different lactobacilli to MMC are different, MMC induction is strain/species-specific to distinct levels of MMC addition (Oliveira et al., 2017. *Front Microbiol*); and some *Lactobacillus* lysogens are not sensitive to MMC, but can be induced using UV treatment or H_2O_2 treatment; Therefore, we cannot determine whether those unresponsive predicted intact prophages are induction failure, inactivated, or false positive one by one. We also cannot determine how many of these "predicted intact prophages" are active. The above contents have been mentioned in the discussion, Line 597-506 Consistently, the identification of active prophages is a difficult issue, and thus, we have not stopped mining and sequencing new *Lactobacillus* phages.

Comment #6: The whole section on CRISPR-Cas system lacks the study of active/inactive CRISPR-Cas system to be then linked to the presence or absence of prophage regions.

Author Reply #6: Thank you for your advice. During the process of CRISPR-Cas systems detection, we have performed manual screening for all predictions. To ensure the integrity and reliability of the predicted CRISPR-Cas system, refer to (Pourcel et al., 2019, *Nucleic Acids Res*), only the CRISPR arrays with the highest confidence level (CRISPR-Cas Finder, level 4) can be reserved. And then we performed Cas gene detection, level 4 CRISPR arrays that have no cas genes clusters were removed. What is left are relatively complete systems, for participating in statistical analysis. Of course, the same as the predicted prophage, we cannot determine whether these CRISPR-Cas systems are active or inactive one by one. Thus, we deleted the part with low-level evidence and re-analyzed, and only reserved the part of the comparison of the number of intact prophages in *Lactobacillus* genomes with or without the CRISPR-Cas system, please see Line 311-331, Fig. 6B and 6C. In addition, in the revised manuscript, to advance our understanding of phage population diversity and bacteria-phage interactions, according to the Commented [A8] raised by Reviewer #3, we added a whole section of Results to introduce our new findings on the association between CRISPR spacers and prophages, including CRISPR spacer clustering, spacer-prophage

alignment, and self-targeting statistics, please see Line 333-363, Fig. 7A, 7B, 7C, and 7D.

Other notes:

Comment #7: Line 43: remove “in”

Author Reply #7: It was our oversight and mistake. Thanks for your revision. It has been deleted.

Comment #8: Line 78: *Lactobacillus* rhamnosus

Author Reply #8: Thanks for your revision. It has been corrected.

Comment #9: Line 124: Remove “etc”

Author Reply #9: Thanks for your revision. It has been deleted.

Comment #10: Lines 131-139: hypothesis based on low level evidence

Author Reply #10: Thank you for your suggestion. It was our thoughtlessness about this section. In the revised manuscript, according to the suggestion of Reviewer #3, instead of focusing on a particular species, we investigated the prophage distribution among all *Lactobacillus* strains which simply divvied into ‘Human/Mammal’ group (*n* = 1022) and ‘Fermented food’ group (*n* = 266). An interesting result was found, strains from the ‘Fermented food’ group tended to harbor a significantly higher number of prophages (Fig. 1C and 1D), for the detailed description, please see Line 137-144. Furthermore, we also put forward hypotheses and inferences about why lactobacilli from fermented food sources carry more prophages in section “Discussion”, please see Line 392-403.

Comment #11: Lines 258-263: rephrase, unclear what the authors want to explain

Author Reply #11: Thank you for your advice. Originally, we would like to investigate the distribution of virulence factors among *Lactobacillus* prophages after the description of ARGs. Although some potential virulence factors were identified, they belong to the genes that enhance bacterial host colonization. As we known, colonization genes of pathogenic bacteria are harmful to host organism, whereas those of *Lactobacillus* are not virulence factors. Thus, we wrote those sentences in the original manuscript. However, to maintain the logical integrity of the revised manuscript, we decided to delete this part that may cause misunderstanding.

Comment #12: Figure 7 is complex specially 7C (data presented cannot be read)

Author Reply #12: Thank you for your suggestion. All three reviewers raised this issue, Fig. 7C actually provides little useful information; thus, we reorganized the manuscript, rewrote most of sentences and removed this figure.

Reviewer #3

General:

Pei and his colleagues have screened for prophage sequences in over 1000 *Lactobacillus* genomes, using software PHASTER. In this paper, the authors reported and characterized the abundant presence of predicted prophage regions in *Lactobacillus* genomes. They detected certain antibiotic resistance genes in the prophage sequences (such as ciprofloxacin resistance in *Lactobacillus plantarum*). They also attempted to correlate the distribution of CRISPR-Cas systems with prophage in *Lactobacillus* genomes. They reported a possible antagonistic relationship between CRISPR type I/III but not type II and the prevalence of intact prophages.

It has been known that prophages are highly prevalent in *Lactobacillus* genomes. However, very little is known regarding its function and relationship with its host. Multiple methods exist to look for prophages in bacterial genomes (such as PHASTER used in this paper). However, most of these tools generate results that can be inconclusive. With that being said, the authors in this paper filtered out the "incomplete or questionable" prophage prediction which was a good practice to clean up the dataset. To my knowledge, this is the first paper that has screened such a large number of *Lactobacillus* genomes to characterize prophage distribution. However, when dealing with a large number of genomes, certain granularity can be lost. I think the authors can dig a bit more on the correlation between prophage and CRISPR. For example, match between spacers and prophage sequence, prophage-mediated anti-CRISPR and etc. Regarding the analyses in this paper, please refer to the manuscript for specific comments.

Author Reply: Thank you very much for your positive comments. Your constructive comments and suggestions have greatly improved the quality of our manuscript, especially for the comments about the linkage between the isolation source and prophage distribution, as well as the CRISPR-prophage association. The following are our responses to every comment.

Commented [A1]: Line 112-114: Please elaborate on the definition of “questionable”, “incomplete”, and “intact”.

Author Reply [A1]: **Thank you for your advice. According to Arndt et al. (Arndt et al., 2019, *Brief Bioinform*) and the instruction of the software, we have added a detailed definition of "intact", "questionable", and "incomplete" in the section "Materials and methods", please see Line 551-566.**

Commented [A2]: I wonder if these predicted intact phages are all active or not. Some data on whether these prophages are indeed inducible or not would be helpful to validate the in silico prediction. You can selectively choose some strains that were predicted to have multiple intact phages, and some strains with incomplete phages and see if the in silico predicted differences actually translate to the bench.

Author Reply [A2]: **Thank you very much for your comments. We previously tried to validate the in silico prediction, 142 strains from 6 different species were selected to induce using Mitomycin C (MMC), and several temperate phages were successfully induced. After enrichment, DNA extraction, sequencing, and alignment, we found that almost all of the induced phages matched the corresponding intact prophage regions predicted by PHASTER (Pei et al., 2020, *Virus Res*). However, the majority of 'potential lysogens' (which carry predicted intact prophages) did not respond to MMC induction (0.70μg/mL). It has been shown that MMC induction is strain/species-specific to distinct levels of MMC addition (Oliveira et al., 2017. *Front Microbiol*); some *Lactobacillus* lysogens are not sensitive to MMC, but can be induced using UV treatment or H_2O_2 treatment; Therefore, we cannot determine whether those unresponsive predicted intact prophages are induction failure, inactivated, or false positive one by one. But at least, we found that almost all of the successfully induced phages were located in the predicted sites of PHASTER, indicating that the reduced dataset of predicted *Lactobacillus* intact prophages may provide a reference for relevant studies and application. The activity of prophage should be carefully evaluated when considering whether *Lactobacillus* lysogens might be used in any fermentation industries or probiotic productions. We have also mentioned the above in the discussion, Line 497-506. Of course, as we said in the manuscript, to promote the field of bacteriophage to cross the technological barrier and then develop rapidly, we are also working on mining/sequencing more active phages.**

Commented [A3]: Line 120: For strains with multiple predicted intact prophage regions, how different or similar in terms of their structures? It seems that authors did not touch on the structure/landscape of the prophages in *Lactobacillus* at all in this paper. Please elaborate on any similarities or differences in prophages regions you have observed among different *Lactobacillus*

species.

Author Reply [A3]: Thank you very much for your valuable suggestions. We neglected to present the structure/landscape of the prophages in *Lactobacillus* in the original manuscript. Combined with the suggestions of you and reviewer #2, to visually and intuitively demonstrate the extent of differences in the structures of *Lactobacillus* prophages for each species or main cluster, we have drawn new figures, please see Fig. 4C and Supplemental Fig. S3B, and added relevant description, please see Line 228-246. Moreover, multiple predicted intact prophage regions within the same strain also showed in Supplemental Fig. S3C.

Commented [A4]: Line 127: Please provide reference for "close genetic relationship"

Author Reply [A4]: Thank you for your advice. We added the reference (Reference No. 26), please see Line 133.

Commented [A5]: Line 127: Based on this study? Please refer to a specific figure or table.

Author Reply [A5]: Thank you for your advice. We rewrote the sentence added a related table (Table 1) at the end, please see Line 135.

Commented [A6]: In this paper, the authors have attempted to look for a correlation between the prophage and origin within a particular species, subsequently reported no such correlation was found. Instead of focusing on a particular species, have the authors investigated if such correlation exists among all *Lactobacillus* strains with predicted intact prophages? Simply group strains based on "human/mammal" and "fermented food" groups (not considering the species), I am curious if you can see a correlation between the isolation source and intact phage distribution that way.

Author Reply [A6]: Thank you very much, that is a good idea! According to your suggestion, we put aside the investigation of particular species and divided 1288 *Lactobacillus* strains with definite isolation sources into the 'Human/Mammal' group (*n* = 1022) and 'Fermented food' group (*n* = 266). The result is interesting, we found that strains from the 'Fermented food' group tended to harbor a significantly higher number of prophages (Fig. 1C and 1D). For a detailed description of the results, please see Line 137-144. Furthermore, we also put forward hypotheses and inferences about why lactobacilli from fermented food sources carry more prophages in section "Discussion", please see Line 392-403.

Commented [A7]: Line 167: Please add a figure to illustrate the landscape of intact prophage for each species. This will give the readers an idea of how different/similar in terms of sizes and structures of prophage regions for each species. This can be included in the Supplemental figures.

Author Reply [A7]: **Thank you very much for your valuable suggestions. We have added two figures to illustrate the landscape of *Lactobacillus* prophages, please see Fig. 4C and Supplemental Fig. S3B-C. For the detailed description of this part, please see the section "Result" Line 228-246.**

Commented [A8]: Line 318-321: Have the authors performed any analysis to check if the sequence of spacer match to any prophage sequences? (Nethery et al., 2019, BMC genomics) reported self-targeting in *Lactbacillus buchneri*. It would be helpful to screen for such events in a large cohort like this. Another interesting aspect would be looking for anti-crispr proteins mediated by prophage sequences. This analysis would indeed shed light on the correlation between CRISPR and prophage in *Lactobacillus*.

Author Reply [A8]: **Thank you very much for your valuable suggestion. We performed analysis on the association between CRISPR spacers and prophages, including CRISPR spacer clustering, spacer-prophage alignment, and self-targeting statistics. In the part of "Results", we added a whole section to introduce our new findings, please see Line 333-363, Fig. 7A, 7B, 7C, and 7D. According to (Nethery et al., 2019, *BMC genomics*), we also discovered that the self-targeting spacers located within intact prophage regions can be observed in 13 of the 16 *Lactobacillus* species, but at a low frequency (52, 3.6%). For the discussion of this part, please see Line 451-458. However, about looking for the anti-CRISPR proteins mediated by *Lactobacillus* prophages, we put all prophage genomes into Anti-CRISPRdb (Dong et al., 2018, *Nucleic Acids Res*, 46: D393-398), but no positive result was found. Thus, further experimental research is needed to identify anti-CRISPR proteins among lactobacilli and their prophages.**

Commented [A9]: Line 330: Figure 7C is very hard to read. Please make it concise and legible or move it to sup figures.

Author Reply [A9]: **Thank you for your suggestion. Fig. 7C provides little useful information; thus, we reorganized the manuscript, rewrote most of the sentences, and removed this figure.**

Commented [A10]: Line 361-366: Again, I agree with the authors that it's highly likely that the distribution of prophages is correlated with the environmental factors. Referring back to the comments made at line 131, I would urge the authors to investigate the correlation of the prophage distribution with origins outside of a particular species.

Author Reply [A10]: **Thank you very much for your positive comment. Please refer to the reply to Commented [A6].**

Commented [A11]: Line 399: Please provide references.

Author Reply [A11]: Thank you for your advice. We rewrote the sentence and added the reference (Reference No. 55), please see Line 444.

Commented [A12]: Line 452: Can authors provide data on the quality of the draft genomes in terms of number of contigs and etc? Please elaborate if the quality of the draft genomes would impact the prophage prediction or not.

Author Reply [A12]: Thank you for your advice. We added the following quality data of each genome in Supplemental Table S1A: 'Genome level', 'Number of Scaffolds/Contigs', and 'Scaf./Ctg. N50 values'.

We have also considered the possibility that the quality and integrity of input genomes impact the accuracy of prophage prediction. We compared the prophage prediction results of different assembly levels of the same strain, take *L. paracasei* FAM18149 and *L. plantarum* ATCC 8014 as examples, as shown in the following figures. The test results show that there was little or no difference in prophage prediction between different assembly levels of the same strain. That is, the higher quality contigs or assembled scaffolds hardly impact the prediction. Of course, assembly draft genomes with exceptionally low quality affect the completeness of the predicted prophage region; thus, to avoid this, on the premise that the number of available genomes of each species is sufficient, we chose the genome with a higher quality of sequencing and assembling as the research object. Among 1,472 *Lactobacillus* genomes used in this study, 59% of them with N50 values > 100 Kb; 81% of them with N50 values > 50 Kb; and 95% of them with N50 values > 30 Kb (please see Supplemental Table S1A). Considering that the genome size of most *Lactobacillus* phages is range 30-50 Kb, we think the quality of the selected genomes in this study could have little effect on the prophage prediction.

***L. paracasei* FAM18149, Complete, GCA_002442835.1**

gi|00000000|ref|NC_000000| Genome; Raw sequence 2969707, gc%: 46.31%

Download summary as .txt file: summary.txt

Total: 8 prophage regions have been identified, of which 3 regions are intact, 2 regions are incomplete, and 3 regions are questionable.

Region	Region Length	Completeness	Score	# Total Proteins	Region Position	Most Common Phage	GC %	Details
CP017261.1,Lactobacillus,paracasei,strain,FAM18149,chromosome,,complete,genome								
1	43Kb	intact	120	56	125666-168700	PHAGE_Lactob_Lrm1_NC_011104(13)	44.10%	Show
2	33Kb	intact	117	45	445604-478620	PHAGE_Lactob_iA2_NC_028830(39)	45.39%	Show
3	13.9Kb	questionable	70	17	652385-666381	PHAGE_Entero_IME_EFm5_NC_028826(3)	44.16%	Show
4	6Kb	incomplete	40	9	741229-747307	PHAGE_Stx2_c_1717_NC_011357(2)	47.51%	Show
5	32.4Kb	intact	150	35	1143913-1176359	PHAGE_Lactob_Lc_Nu_NC_007501(12)	42.95%	Show
6	55.2Kb	incomplete	40	66	1531383-1586667	PHAGE_Lactob_PLE3_NC_031125(24)	44.99%	Show
7	16.2Kb	questionable	90	19	1673858-1690100	PHAGE_Bacter_Diva_NC_028788(2)	47.63%	Show
CP017262.1,Lactobacillus,paracasei,strain,FAM18149,plasmid,pFAM18149.21,,complete,sequence								
8	27.9Kb	questionable	80	32	25558-53504	PHAGE_Staphy_SPbeta_like_NC_029119(2)	42.97%	Show

***L. paracasei* FAM18149, Scafford, GCA_003712485.1**

Total: 8 prophage regions have been identified, of which 3 regions are intact, 2 regions are incomplete, and 3 regions are questionable.

Region	Region Length	Completeness	Score	# Total Proteins	Region Position	Most Common Phage	GC %	Details
LKFR01000002.1,Lactobacillus,paracasei,strain,FAM18149,FAM18149_scaffold_0002,,whole,genome,shotgun,sequence								
1	27.9Kb	questionable	90	26	8890-36836 ⓘ	PHAGE_Staphy_SPbeta_like_NC_029119(2)	42.97%	Show ⓘ
LKFR01000006.1,Lactobacillus,paracasei,strain,FAM18149,FAM18149_scaffold_0006,,whole,genome,shotgun,sequence								
2	33Kb	intact	120	46	15650-48667 ⓘ	PHAGE_Lactob_iA2_NC_028830(40)	45.40%	Show ⓘ
3	13.9Kb	questionable	70	17	222432-236428 ⓘ	PHAGE_Entero_IME_EFm5_NC_028826(3)	44.16%	Show ⓘ
LKFR01000007.1,Lactobacillus,paracasei,strain,FAM18149,FAM18149_scaffold_0007,,whole,genome,shotgun,sequence								
4	6Kb	incomplete	40	9	39712-45790 ⓘ	PHAGE_Stx2_c_1717_NC_011357(2)	47.52%	Show ⓘ
5	32.4Kb	intact	150	35	442396-474842 ⓘ	PHAGE_Lactob_Lc_Nu_NC_007501(12)	42.95%	Show ⓘ
LKFR01000008.1,Lactobacillus,paracasei,strain,FAM18149,FAM18149_scaffold_0008,,whole,genome,shotgun,sequence								
6	55.2Kb	incomplete	40	66	35806-91090 ⓘ	PHAGE_Lactob_PLE3_NC_031125(24)	44.99%	Show ⓘ
7	16.2Kb	questionable	90	19	178281-194523 ⓘ	PHAGE_Bacter_Diva_NC_028788(2)	47.63%	Show ⓘ
LKFR01000010.1,Lactobacillus,paracasei,strain,FAM18149,FAM18149_scaffold_0010,,whole,genome,shotgun,sequence								
8	42.5Kb	intact	120	57	233730-276326 ⓘ	PHAGE_Lactob_Lrm1_NC_011104(13)	44.14%	Show ⓘ

***L. plantarum* ATCC 8014, complete, GCA_002749655.1**

>CP024413.1 Lactobacillus plantarum strain ATCC 8014 chromosome, complete genome

Download summary as .txt file: summary.txt

Total: 4 prophage regions have been identified, of which 2 regions are intact, 1 regions are incomplete, and 1 regions are questionable.

Region	Region Length	Completeness	Score	# Total Proteins	Region Position	Most Common Phage	GC %	Details
1	16.9Kb	questionable	70	23	37804-54802 ⓘ	PHAGE_Strept_315.2_NC_004585(3)	42.37%	Show ⓘ
2	38.7Kb	intact	140	53	512877-551634 ⓘ	PHAGE_Lister_B025_NC_009812(9)	41.51%	Show ⓘ
3	42.7Kb	intact	140	53	1020394-1063100 ⓘ	PHAGE_Lactob_Sha1_NC_019489(19)	41.50%	Show ⓘ
4	16.1Kb	incomplete	20	7	1093207-1109313 ⓘ	PHAGE_Entero_phi92_NC_023693(4)	41.24%	Show ⓘ

***L. plantarum* ATCC 8014, contig, GCA_002370965.1**

gi|00000000|ref|NC_000000| Genome; Raw sequence 3254764, gc%: 44.50%

Download summary as .txt file: summary.txt

Total: 3 prophage regions have been identified, of which 2 regions are intact, 0 regions are incomplete, and 1 regions are questionable.

Region	Region Length	Completeness	Score	# Total Proteins	Region Position	Most Common Phage	GC %	Details
NZ_LJDC01000009.1,Lactobacillus,plantarum,strain,ATCC,8014,EV52_contig000009,,whole,genome,shotgun,sequence								
1	42.7Kb	intact	150	54	261416-304122 ⓘ	PHAGE_Lactob_Sha1_NC_019489(19)	41.50%	Show ⓘ
NZ_LJDC01000014.1,Lactobacillus,plantarum,strain,ATCC,8014,EV52_contig000014,,whole,genome,shotgun,sequence								
2	16.8Kb	questionable	90	25	21003-37872 ⓘ	PHAGE_Strept_315.2_NC_004585(3)	42.43%	Show ⓘ
NZ_LJDC01000027.1,Lactobacillus,plantarum,strain,ATCC,8014,EV52_contig000027,,whole,genome,shotgun,sequence								
3	38.7Kb	intact	130	61	114-38871 ⓘ	PHAGE_Lister_B025_NC_009812(9)	41.51%	Show ⓘ

Commented [A13]: Line 454: Please provide more details on how you prepared DNA extraction, and how you grow the bacteria. Did you perform quality check on the resulting DNA sequences? Trimming, assembly?

Author Reply [A13]: Thank you for your advice. We added the description for bacterial culture and genomic DNA extraction in the section "Materials and methods" (please see Line 532-537). Indeed, the raw data of Illumina Hiseq contain some low-quality data, therefore, we performed quality trimming on the raw data as follow steps: (1) Remove the adapter sequence in reads; (2) Cut and remove the bases other than A, G, C, and T at the 5' end; (3) Trim the ends of reads with lower sequencing quality (the sequencing quality value is less

than Q20); (4) Remove the reads that contain 10% of N bases; (5) Discard the adapter and the small fragments whose length is less than 25 bp after quality trimming. The high-quality reads obtained after the above series of quality trimming were used for genome assembly. We also detailed the description for genome sequencing and draft assembly in Line 538-546.

Commented [A14]: Line 463: What's considered as intact, incomplete and questionable? Any manual curation and validation on prophage regions that were predicted as intact?

Author Reply [A14]: **Thank you for your comments. The detailed definition of "intact", "questionable", and "incomplete" was provided in Line 551-566. All 1,459 predicted intact prophages were verified for the presence of cornerstone genes (five core modules: Lysogeny, DNA replication, DNA packaging, Morphogenesis, and Lysis), 81% (1,183/1,459) of them have all five core modules' genes; 97% (1,420/1,459) of them have four or more core modules' genes. A small number of predicted intact prophages were annotated with a few known functional genes, but a large number of phage-like proteins. Therefore, we consider that most of these predicted intact prophages have relatively complete genomic organizations. As for whether these predicted intact prophages in this study can be induced and transformed on the bench, please refer to our reply on Commented [A2].**

Commented [A15]: Line 485: Please provide reference.

Author Reply [A15]: **Thank you for your advice. We have added the reference (ISO 10932:2010, References No. 74), please see Line 584.**

Commented [A16]: Line 486: Please provide more details on how you determined the MIC. For example, which media did you use? How did you prepare the bacteria culture?

Author Reply [A16]: **Thank you for your advice. We have updated the manuscript to include detailed description on the methods of antibiotic susceptibility testing, including bacterial culture medium, culture conditions, concrete operation method, and related references. For details, please see Line 581-595.**

In addition, thank you very much for your revision on our manuscript in terms of language, wording, sentence structure, etc. (in Line 33, Line 43, Line 46, Line 53, Line 98, Line 116, Line 159-160, Line 348, Line 359, Line 405-408, Line 419-420, Line 422-424, Line 429-430, Line 434-436). We have adopted all of them in the revised manuscript.

Thank you again for your detailed, meticulous, excellent editorial work on the manuscript. We also thank three reviewers for your constructive, careful, and valuable scientific comments to strengthen our manuscript. I hope these responses and this revised manuscript are acceptable to you.

Thank you and best regards.

Yours sincerely,

Wei Chen

School of Food Science and Technology, Jiangnan University

Wuxi, No. 1800 Lihu Avenue, Jiangsu, 214122, P. R. China

Phone number: 86-510-85912155

Fax number: 86-510-85912155

Email address: chenwei66@jiangnan.edu.cn

May 11, 2021

Prof. Wei Chen
Jiangnan University
1800 Lihu Ave, Wuxi, Jiangsu 214122, P.R. China.
Wuxi, Jiangsu 0510
China

Re: mSystems01211-20R1 (Comprehensive scanning of prophages in *Lactobacillus*: distribution, diversity, antibiotic resistance genes, and linkages with CRISPR-Cas systems)

Dear Prof. Wei Chen:

Your manuscript has been accepted, and I am forwarding it to the ASM Journals Department for publication. For your reference, ASM Journals' address is given below. Before it can be scheduled for publication, your manuscript will be checked by the mSystems senior production editor, Ellie Ghatineh, to make sure that all elements meet the technical requirements for publication. She will contact you if anything needs to be revised before copyediting and production can begin. Otherwise, you will be notified when your proofs are ready to be viewed.

As an open-access publication, mSystems receives no financial support from paid subscriptions and depends on authors' prompt payment of publication fees as soon as their articles are accepted. You will be contacted separately about payment when the proofs are issued; please follow the instructions in that e-mail. Arrangements for payment must be made before your article is published. For a complete list of **Publication Fees**, including supplemental material costs, please visit our website.

Corresponding authors may join or renew ASM membership to obtain discounts on publication fees. Need to upgrade your membership level? Please contact Customer Service at Service@asmusa.org.

For mSystems research articles, you are welcome to submit a short author video for your recently accepted paper. Videos are normally 1 minute long and are a great opportunity for junior authors to get greater exposure. Importantly, this video will not hold up the publication of your paper, and you can submit it at any time.

Details of the video are:

· Minimum resolution of 1280 x 720
· .mov or .mp4. video format
· Provide video in the highest quality possible, but do not exceed 1080p
· Provide a still/profile picture that is 640 (w) x 720 (h) max

We recognize that the video files can become quite large, and so to avoid quality loss ASM suggests sending the video file via https://www.wetransfer.com/. When you have a final version of

the video and the still ready to share, please send it to Ellie Ghatineh at eghatineh@asmusa.org.

Thank you for submitting your paper to mSystems.

Sincerely,

Lee Ann McCue
Editor, mSystems

Journals Department
American Society for Microbiology
1752 N St., NW
Washington, DC 20036
E-mail: peerreview@asmusa.org
Phone: 1-202-942-9338

Fig. S3: Accept
Table S4: Accept
Table S1: Accept
Fig. S2: Accept
Table S2: Accept
Table S3: Accept
Data Set: Accept
Fig. S1: Accept
Fig. S4: Accept
